# From the Balkan Peninsula to the Mesic Grassland Areas of Central Europe: Morpho-Genetic Diversity and Niche Differentiation in the Allopolyploid Complex of the Austrian Speedwell

**DOI:** 10.3390/plants15060955

**Published:** 2026-03-20

**Authors:** David Jiménez-García, Noemí López-González, Daniel Pinto-Carrasco, Nélida Padilla-García, Santiago Andrés-Sánchez, Blanca M. Rojas-Andrés, M. Montserrat Martínez-Ortega

**Affiliations:** 1 Área de Botánica, Universidad de Salamanca, 37007 Salamanca, Spain; noe_lg@usal.es (N.L.-G.); dpintocarrasco@usal.es (D.P.-C.); nelidam@usal.es (N.P.-G.); santiandres@usal.es (S.A.-S.); rojasabm@usal.es (B.M.R.-A.); 2 Herbario y Biobanco de ADN Vegetal, Universidad de Salamanca, 37007 Salamanca, Spain

**Keywords:** allopolyploidy, Balkan Peninsula, colonization patterns, grassy habitats, intraspecific diversity, speciation, *Veronica austriaca*

## Abstract

The Balkan Peninsula is a biodiversity hotspot where topographic and habitat heterogeneity have shaped genetic differentiation. Polyploidization significantly contributes to diversification within plant lineages, including the allopolyploid complex of the Austrian speedwell, which comprises diploid, tetraploid and hexaploid lineages. We sampled 751 individuals from 50 populations belonging to this complex across the Balkan Peninsula and Central Europe. Diversity patterns were investigated through microsatellite markers (SSRs), plastid DNA sequences, ploidy estimations, morphological data and climatic niche differentiation analysis. Five lineages were detected within the complex according to nuclear DNA data. The plastid DNA haplotypes form two main groups that overall match those detected by SSR data and could suggest that the hexaploid lineage resulted from two different allopolyploid events. The hexaploid shows higher nuclear genetic diversity and morphological variation than its lower-ploidy relatives, which might allow the species to respond to a wider range of environmental conditions and be responsible for its success (i.e., a broader geographic range and ecological niche). Style length is a crucial character to distinguish diploids from polyploids, which may affect pollination biology within the complex.

## 1. Introduction

The Balkan Peninsula has long been acknowledged as a major center of biodiversity [1]. With ca. 6500 species of vascular plants reported, the region is more species-rich than any area of comparable extension within Europe [2]. Among the factors responsible for this amazing species richness are that it is a topographically and climatically diverse region with high geological complexity [3,4], in addition to the fact that it is placed at the junction of three continental regions representing a crossroads of several major floras [5]. The numerous mountain formations display a complex pattern of small blocks geographically close to each other [6], and the large habitat heterogeneity—among those, mesic meadows and other grassland areas, such as prairies and drier steppe-like environments—have led to genetic differentiation among taxa, resulting also in a hotspot of endemism [7]. The mainly lowland areas that facilitated the persistence of species during past climatic oscillations may have acted as ‘museums’ for the conservation of diversity, as ‘cradles’ of new diversity (i.e., regional biodiversity hotspots; [8,9]) and may have also provided corridors allowing migrations of plant species from grassland habitats.

Despite its species-richness, its importance as a centre of diversification, and its role as a major source of post-glacial colonization of Central and Northern Europe, not much is known about the patterns of diversity and phylogeography of biota from the Balkans, as compared to the Italian and Iberian peninsulas. Many groups of organisms remain unstudied [10] and detailed studies centered in the Balkan Peninsula have mostly put their focal point on vertebrates (and especially herpetofauna; e.g., [11,12]), butterflies (e.g., [13]) or alpine plants (e.g., [14,15]). In contrast, only a few studies (e.g., [16]) have focused on nowadays widely distributed plant species representative of dry to mesic grassland habitats from Europe. These environments, which are potentially common in Eurasia, have been traditionally subjected to high pressure mainly from agriculture and forestry and suffer many times from severe fragmentation. Therefore, additional studies are necessary to better achieve their conservation.

Among the different mechanisms involved in diversification, polyploidization has played a major role in the evolution of many taxa within the Balkan Peninsula (e.g., [17,18]). Polyploidization has been proposed to have facilitated the colonization of new niches available after the climatic oscillations of the Quaternary and would therefore be involved in the post-glacial expansion of some species (or cytogenetic units) during the Holocene [19,20]. Particularly, allopolyploidy has been proposed as a major mode of speciation (e.g., [21]) and is believed to favour both niche (e.g., [22]) and, at least in particular cases [23,24], range expansion (but see [25]). Although niche expansion of polyploids with respect to their diploid progenitors is not a consistent trend across species in polyploid evolution [26], it is supported by many empirical studies [27,28,29,30,31]. Several studies utilizing novel methods—among them, Species Distribution Models (SDMs)—(e.g., [32]) have indicated that the ecological niches of allopolyploids often differ from at least one of their lower ploidy ancestors (e.g., [33]). In contrast, other investigations suggest that allopolyploids may occupy intermediate or non-divergent ecological niches (e.g., [34]). Consequently, additional studies are necessary to interpret which is the role that niche shift plays in the establishment of polyploid lineages.

Species are often used as the basic units of analysis in biogeography. However, species can be described as an assemblage of genetic lineages varying in their genetic inter-relationship and spatial distribution [35]. Thus, addressing intraspecific genetic variation in widely distributed species within an ecogeographic context is necessary to understand key topics in ecology, evolution and biogeography [36]. Widespread species from common environments provide opportunities to study both genetic and phenotypic variation patterns across ample ranges of climatic conditions. Determining these patterns becomes a crucial preliminary step to design studies specifically aimed at understanding the genomic basis underlying local adaptation in climate-related traits [37].

Here, the geographically widespread allopolyploid complex of the Austrian speedwell (with a base chromosome number of *x* = 8 [38]) was selected to investigate whether the combination of subgenomes in an allopolyploid species may yield plants with increased genetic and morphological diversity, as well as enhanced colonization abilities. The Austrian speedwell complex is composed of suffrutex perennial herbs that occur in vast regions from Central and Eastern Europe and Asia. Taxonomically, it belongs to *Veronica* subsect. *Pentasepalae* Benth., and comprises the diploid *V. dalmatica* Padilla-García, Rojas-Andrés, López-González and M.M.Mart.Ort., an uncertain morphologically cryptic tetraploid entity and the hexaploid *V. austriaca* L.

In this study, the species name *V. austriaca* is applied according to the last available taxonomic treatment [39], with the modifications introduced by the results derived from Padilla-García et al. [40] and López-González et al. [41], which means that *V. austriaca* includes only hexaploids. While many narrow endemics can be found within *Veronica* subsect. *Pentasepalae* [40,42], only a few species—among them *V. austriaca*—are widely distributed. Furthermore, *V. austriaca* displays wide ecological amplitude and is therefore present in a variety of habitats, being mostly represented in grassland areas (from mesic meadows to Mediterranean dry steppe-like environments) and sometimes in grassy forest glades or forest edges. It also extends to a wide array of climatic and altitudinal (60–2000 m) conditions all throughout the Balkan Peninsula, Central Europe, and—according to Borissova [43]—it is also present in Belarus, European Russia, Moldavia, Ukraine and S Caucasus.

In addition to the wide ecological preferences, *V. austriaca* displays high morphological diversity. Thus, several infraspecific taxa [usually three subspecies, i.e., *V. austriaca* subsp. *austriaca*, *V. austriaca* subsp. *dentata* (F.W. Schmidt) Watzl and *V. austriaca* subsp. *jacquinii* (Baumg.) Watzl] have been described within the variation in this taxonomically challenging species, which can be distinguished based on foliar traits. There is, for example, an apparent gradient regarding leaf incision (although statistically significant differences have been found to separate the three subspecies recognized within *V. austriaca* [44]) that has been interpreted by different authors in different ways, with the description of the individuals that show intermediate character states either as “transitional forms” between subspecies or as taxonomic entities of hybrid origin [45,46]. Recently, it has turned out that some of these populations from the Western Balkans that show intermediate character states are the result of allopolyploid events [40]. Specifically, in the formation of the hexaploid *V. austriaca* (subsp. *jacquinii*), the diploid species *V. dalmatica* and a scientifically still unnamed “uncertain tetraploid” have been involved, with the possibility of recent gene flow among these different cytotypes [41]. Thus, both taxonomic entities (i.e., 2*x* and 4*x* lineages), together with the hexaploid *V. austriaca*, shape the allopolyploid complex which is the object of this study.

Thus, we here investigate the diversity patterns of the Austrian speedwell allopolyploid complex across its range in the Balkan Peninsula and Central Europe. For this, a combined approach is applied using ploidy levels estimated by flow cytometry (FCM), data from nuclear markers (Simple Sequence Repeats, SSRs) and plastid DNA (cpDNA) sequences. Phenotypic and ecological variation are also considered, respectively, through the morphometric analysis of several leaf and fruit characters, through the prediction of the ecological niche optimum and breadth of the members of the allopolyploid complex, as well as the prediction of the present potential distribution areas of the hexaploid *V. austriaca* using SDMs. The specific goals are: (1) to assess the genetic structure and diversity in *V. austriaca* and its putative diploid and tetraploid ancestors; (2) to describe the morphological variability and ecological niche differentiation of the members of the allopolyploid complex; (3) to understand the historical processes that may have been responsible for their range expansion from the Balkan Peninsula, (4) to discuss the impact that allopolyploidy may have had in the colonization abilities and expansion of a species that is nowadays widely distributed in the mesic grassland areas of Europe.

## 2. Materials and Methods

### 2.1. Plant Material

**Figure 1:**
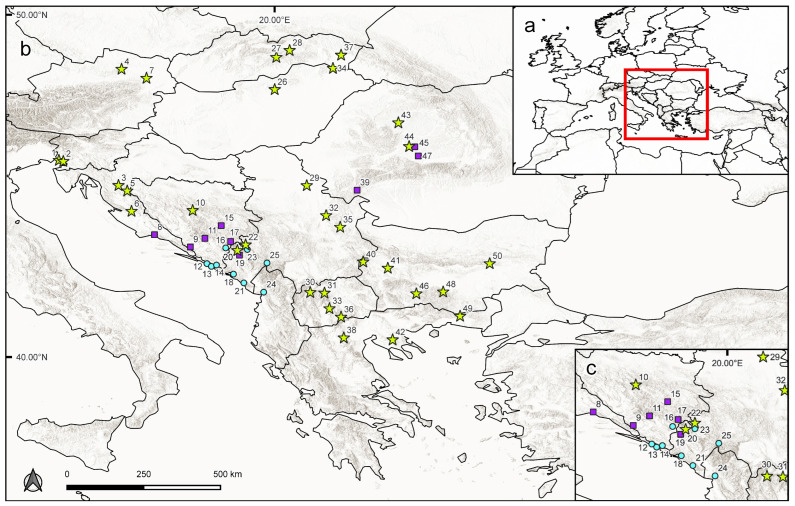
Map showing sampling localities and ploidy levels estimated for the *Veronica* populations included in this study. (**a**) Location of the study area in Europe; (**b**) main study area; (**c**) detail of the studied area corresponding to Bosnia and Herzegovina, Montenegro, southern Croatia and northern Albania. Turquoise circles refer to diploid populations (*V. dalmatica*), purple squares to tetraploid ones (“uncertain tetraploids”), while pistachio-colored stars represent hexaploid populations (*V. austriaca*). Locality numbers refer to those in Table S1.

Details about the location and taxonomic assignment of the samples included are provided in Table S1. The spatial distribution of the selected populations is displayed in Figure 1. Fresh leaf material was collected and stored in silica gel. The complete dataset comprises 751 individuals corresponding to 50 populations. A mean of 15 (from 4 to 20) individuals per population were sampled whenever possible, given that many times populations were very small. Initial plant identification was based on the most recent taxonomic treatment by Rojas-Andrés and Martínez-Ortega [39] with modifications from Padilla-García et al. [40] and López-González et al. [41]. Vouchers were deposited in the herbarium SALA (abbreviation according to Thiers [47], continuously updated).

### 2.2. Laboratory Procedures

#### 2.2.1. DNA Ploidy Level Estimations

DNA ploidy levels were estimated by flow cytometry (FCM) from silica gel-dried leaves. For the present study, measurements on 456 individuals were newly generated; the remaining estimations were obtained from López-González et al. [41]. Nuclear suspensions were prepared following the method described by Galbraith et al. [48]. Leaf tissue of either one individual or two to six individuals was chopped together with leaf tissue from an internal standard using a sharp razor blade in a Petri dish with Woody Plant Buffer solution with slight modifications [49]. Several internal standards were employed depending on the C-value and standard availability: *Raphanus sativus* L. (2C = 1.11 pg; [50]), *Solanum lycopersicum* L. (2C = 1.96 pg; [50]), *Solanum pseudocapsicum* L. (2C = 2.59 pg; [51]), *Zea mays* L. cv. ‘CE-777’ (2C = 5.43; [52]), *Pisum sativum* L. cv. ‘Kleine Rheinländerin’ (2C = 8.84; [53]), and *Pisum sativum* cv. ‘Ctirad’ (2C = 9.09; [54]). Nuclear suspensions were filtered through a 48 μm nylon mesh, incubated with RNase and stained with a saturating solution of propidium iodide (PI) following Loureiro et al. [49] and Rojas-Andrés et al. [55].

For each individual, one run of 5000 counts was performed on a CyFlow Space (Partec GmbH, Münster, Germany; equipped with a 532 nm solid-state laser). Results were acquired through the Partec FloMax software v2.4d (Partec GmbH, Münster, Germany). DNA ploidy level was estimated for each sample based on the C-values and the available chromosome counts for the studied species [38,56].

#### 2.2.2. DNA Extraction

Total genomic DNA was extracted from silica-gel-dried material following the CTAB protocol [57] with slight modifications. For each individual, 20–25 mg of dried leaves was used. The quality of the extracted DNA was checked on 1% TAE-agarose gels, and the amount of DNA was estimated using a Nanodrop 2000C Spectrophotometer (Thermo Scientific, Waltham, MA, USA). DNA extractions are deposited at the Biobanco de ADN Vegetal of the University of Salamanca (Spain).

#### 2.2.3. SSR Amplification, Fragment Analysis and Genotyping

In order to assess the nuclear genetic diversity of the studied taxa, twelve SSR polymorphic primer pairs were employed (see Table 1 in López-González et al. [58]) to genotype the 751 individuals corresponding to the whole dataset. Following the procedure developed by Schuelke [59], the sequence-specific forward primers were marked at the 5′ end with an M13 tail (5′-TGTAAAACGACGGCCAGT-3′) (Eurofins , Ebersberg, Germany) and then labeled with 5-FAM, VIC, NED, or PET fluorescent dyes (see Table 1 in [58]) (Life Technologies, Carlsbad, CA, USA). PCR conditions were as follows: an initial step at 94 °C for 2 min followed by 35 cycles of 1 min at 94 °C, 1 min at 50–58 °C, and 50 s at 72 °C; 10 cycles of 1 min at 94 °C, 1 min at 53 °C, and 50 s at 72 °C; and a final extension of 15 min at 72 °C. Reaction volumes (total volume: 15 μL) included 50 ng of DNA template, 5 μL 5×Green GoTaq Reaction Buffer (Promega, Madison, WI, USA), 0.2 mM of each dNTP, 0.16 mM of each reverse and fluorescent-labeled M13 primer, 0.04 mM of forward primer and 0.75 units GoTaq DNA Polymerase (Promega). All PCR reactions were performed on a Mastercycler-Pro thermocycler (Eppendorf, Hamburg, Germany). PCR products were visualized on 2.5% TBE-agarose gels and multiplexed for genotyping. Fragment analysis was conducted at the Unidad de Genómica-Campus Moncloa (Universidad Complutense de Madrid, Madrid, Spain) using the internal GeneScan 500 LIZ Size Standard (Applied Biosystems, Waltham, MA, USA) in a multi-capillary sequencer ABI Prism 3730 (Applied Biosystems). Genotyping was performed through GeneMarker Software version 1.8 (SoftGenetics, State College, PA, USA), and the peaks were scored manually.

Reproducibility tests were performed over 5–10% of the total number of individuals. Two to three independent runs at different times were carried out. The outcomes indicated 95% match in the results, suggesting that the microsatellite analyses are highly reproducible.

#### 2.2.4. Plastid DNA Amplification and Sequencing

The plastid DNA regions *trnH*-*psbA* and *ycf6*-*psbM* were amplified for 312 selected individuals from the total dataset (4 to 13 individuals per population). From these, 176 sequences of each region were newly generated, while the remaining ones were obtained from Rojas-Andrés et al. [55] or from López-González et al. [41]. These plastid regions were chosen following Rojas-Andrés et al. [55] due to their high levels of variability. The *ycf6*-*psbM* spacer was amplified using the *ycf6F* forward and *psbMR* reverse primers [60], and the *trnH*-*psbA* spacer with the forward primer *psbA* [61] and the reverse primer *trnH*2 [62]. PCR conditions for the amplification consisted of an initial denaturation cycle of 2 min at 95 °C followed by 40 denaturation cycles of 30 s at 95 °C, annealing for 30 s at 55 °C, and extension for 2 min 30 s at 72 °C, followed by a final extension phase of 10 min at 72 °C. The reaction volumes (total volume: 25 μL) included: 36 ng of DNA template, 5 μL 5×Green GoTaq Reaction Buffer (Promega), 0.2 mm dNTPs, 0.3 μm each primer and 0.85 units GoTaq DNA Polymerase (Promega). All PCR reactions were performed on an Eppendorf-Mastercycler-Pro thermocycler. All amplified fragments were visualized on 1% TBE-agarose gels and purified with ExoSap-IT (USB Corporation, Cleveland, OH, USA) following the manufacturer’s instructions. PCR products were sequenced by Macrogen Inc. (Seoul, Republic of Korea) using an ABI Prism 3730XL DNA analyser (Applied Biosystems). The accession numbers of the sequences obtained from Rojas-Andrés et al. [55] are KT361722–KT361723, KT361726–KT361728, KT361731–KT361733, KT361775, KT361778–KT361780, and KT361783–KT361785 (*trnH-psbA* and *ycf6-psbM*, respectively). The accession numbers of the sequences obtained from López-González et al. [41] are MN997128–MN997307 (*trnH-psbA*) and MT084169–MT084346 (*ycf6-psbM*). The newly generated sequence data were deposited in the European Nucleotide Archive (ENA) at EMBL-EBI under the project accession numbers PRJEB102049 (OZ360793–OZ360968) (*trnH-psbA*) and PRJEB102053 (OZ360969–OZ361144) (*ycf6-psbM*).

### 2.3. DNA Data Analyses

Given that genotyping of SSRs with hexaploids shows a remarkable drawback due to the difficulty of distinguishing the exact number of copies for a given allele [63], the scoring was done indicating the alleles that were present in each individual, and the rest of the copies were coded as missing data. The summary statistics were calculated using the original matrix (individuals × allele length) with SPAGeDi [64]. Given that this software reported 88.4% missing data globally (across all individuals and loci), which may prevent an accurate assessment of genetic diversity, a presence/absence matrix was generated to calculate this parameter with AFLPdiv 1.1 [65] and Popgene v. 1.32 [66]. Spatial distribution of genetic diversity was represented using ‘sf’, ‘rnaturalearth’, ‘rnaturalearthdata’ and ‘ggplot2’ packages in R [67,68,69,70], and the diversity boxplots were represented using ‘ggplot2’ and ‘tidyr’ packages in R [70,71].

To infer population structure and assign individuals to populations based on the SSR genotypes, a Bayesian clustering analysis based on the MCMC algorithm was performed using Structure v.2.3.4 [72], with the original matrix as an input. Multiple runs of Structure were performed by setting *K* from one to ten. In order to reach convergence, a burn-in of 1 × 10^6^ iterations was applied, followed by 1 × 10^6^ MCMC iterations for each run, and 10 runs were done for each value of K. To determine the most probable value of *K*, StructureSelector [73] was used following the criterion described by Evanno et al. [74]. The results of independent Structure runs were summarized and visualized using both StructureSelector and CLUMPAK (Cluster Markov Packager Across K) [75]. A map with bar plots displaying the probability of belonging to a cluster (by population) was elaborated using the software *ArcMap* v. 10.6 [76].

To complement this approach and considering that it is not possible to determine whether the populations under study follow the Hardy–Weinberg equilibrium model, the genetic structure was additionally investigated using the non-hierarchical *K*-means clustering [77], which does not assume this equilibrium. This clustering was performed using the R script of Arrigo et al. [78], and the optimal number of genetic clusters was calculated using the method of Evanno et al. [74] as adapted in Arrigo et al. [78].

Additionally, to visualize differentiation among possible genetic groups a Discriminant Analysis of Principal Components (DAPC; [79]) was performed, first, for both the complete dataset and then−given that it was one of our aims to explore the expansion of this species−focused on the hexaploid *V. austriaca*, using the ‘adegenet’ package in R [80] with no *a priori* assignment of individuals to groups and using the presence/absence matrix (excluding monomorphic alleles in the hexaploids) as an input. A minimum-spanning tree connecting the cluster centroids based on the squared distances among populations was superimposed.

Regarding the plastid DNA sequence data, the alignment was carried out using SATé [81] and visualized in Geneious Pro v. 6.0.6. (Biomatters). Gaps and non-informative variable sites were removed with Gblocks0.91b [82], keeping the default parameters. Inversions were found in the analysed regions and were subsequently removed from the sequences. Mononucleotide repeats of different sizes were excluded because they seem to be prone to homoplasy at large geographical scales [83]. Plastid DNA sequences were used to construct an unrooted haplotype network. The statistical parsimony algorithm [84] was applied to infer the genealogical relationships among haplotypes through the TCS 1.21 software [85]. The ambiguities encountered in the haplotype network, which are probably due to homoplasy (recurrent mutations) and/or recombination within DNA regions [86], were resolved by following the guidelines in Crandall and Templeton [87], based on the frequency of appearance of the different alleles and the geographic location of the samples involved.

### 2.4. Morphometrics Analyses

Five fruit characters and twenty leaf characters (abbreviations shown in Table S2) were measured for all 50 populations included in this study. Fruit characters were measured in three specimens per population, and five fruits per specimen were considered. Leaf characters were measured in three specimens per population, except in cases in which the available material was insufficient. Leaf measurements were taken from two different leaves: one situated in the central segment of the stem (medium leaf) (see Figure 2 in Andrés-Sánchez et al. [88] for further details) and another one in the apical shoot (see Figure 3 in [88]).

Leaf measurements, except those related to the indumentum, were taken with a digital electronic caliper Digimatic 500 (Mitutoyo American Corporation, Aurora, CO, USA). One measurement was made for each variable except for hair length, for which five trichomes per leaf were considered. Hair “density” was indirectly estimated by counting the number of hairs on 1 cm-long linear transects at the leaf margin and at the medium part of the leaf. Hair length, “density” and fruit measurements were determined by means of a stereoscopic zoom microscope NIKON SMZ-U (Nikon Corporation, Tokyo, Japan) equipped with a video camera SONY 3CCD DXC-930P (Sony Corporation, Tokyo Japan). The photos taken were transferred to a computer and examined through the image-analysis software Image-Pro Plus version 1.0 (Media Cybernetics Inc., Rockville, USA). In an effort to avoid the size effect, some characters were considered as quotients. Arithmetic means were calculated and—considering that previous results had already demonstrated differences in foliar traits among the subspecies recognized within *V. austriaca* [44], as well as the necessity to explore further floral characters to aid in distinguishing the species with pinnatisect leaves (i.e., *V. dalmatica* from *V. austriaca* ssp. *jacquinii*) [40]—variables were assessed individually in a visual exploration through boxplots considering different groupings in order to explore differences among ploidy levels among the genetic-geographic groups obtained through the Bayesian analysis and DAPC of SSR data (see Results) within the hexaploid species *V. austriaca* and between the cryptic taxa *V. dalmatica* and *V. austriaca* ssp. *jacquinii*. To analyze morphological characters, we first verified the assumptions of normality and homogeneity of variances using Shapiro–Wilk and Levene’s tests, respectively. Since the data met parametric assumptions, we performed a one-way ANOVA test. To account for multiple comparisons and control the Type I error rate, post hoc differences between groups were identified using Tukey’s Honestly Significant Difference (HSD) test. The boxplots and their corresponding ANOVA tests were calculated using ‘ggplot2’ and ‘car’ packages in R [70,89].

### 2.5. Analyses Based on Climatic Variables

The localities used for these analyses were only those visited by the authors, where the presence of the relevant taxonomic entity was confirmed. Geographical coordinates were collected during fieldwork using GPS.

#### 2.5.1. Species Distribution Models (SDMs)

SDMs were performed to investigate present niche availability for the three genetic-geographic groups detected by SSRs within the hexaploid *V. austriaca.* Values were extracted from the layers based on the *V. austriaca* occurrences with the function ‘extract’ of the R package ‘raster’ [90]. Spatial resolution was 2.5 arc-minutes for all of them. The layers were trimmed around the occurrences of *V. austriaca* in the study area (extent: 10° N, 35° W to 30° N, 50° W) using ‘raster’ [90]. Each layer was converted to BIL format. The columns (i.e., environmental variables) of this primary matrix were checked for high (>|0.80|) pairwise correlation. Variables lacking high correlation values were retained. The layers were separated into groups according to correlation values to be able to sequentially select one of each group in further steps. For the GLMs, occurrences were classified according to the molecular groups obtained by Bayesian clustering and DAPC. Background points were generated through the formula ‘randomPoints’ included in the ‘dismo’ R package [91]. The background points were generated across the entire range to provide a comparative data set to be contrasted against the occurrences. The final set of variables for each molecular group was selected after applying the variance inflation factor values (VIF; [92]) over the models. These analyses were performed through the ‘vif’ function of the ‘HH’ package [93] to test for the absence of multicollinearity.

Once the set of variables was determined for each hexaploid genetic-geographic group, final models were performed combining GLMs with three frequently used modelling techniques: random forest (RF; [94]), artificial neural networks (ANNs; [95]), and maximum entropy (ME; [96]). Recent studies have suggested that machine-learning methodology may perform better than the traditional regression-based algorithms [97]. RF, ANNs and ME are considered to be among the most powerful machine learning algorithms for ecological prediction [98,99,100] and for obtaining powerful ensemble models [101,102]. Predictions from single SDMs are commonly highly variable and unreliable, while ensemble approaches could yield more accurate predictions [103].

Modelling with RF, ANN and GLM was performed using the R package ‘Biomod2’ [104] and for the ME algorithm the ‘ENMeval’ library was employed [105]. RF, ANN and GLM models were carried out using a randomly chosen 75% of the data, with the remaining 25% used for cross-validation to assess the performance of each model using the area under the receiver operator curve (AUC-ROC) assessment criteria. Five replicates were run for each model. The contribution of each variable was calculated as a weighted average of the AUC-ROC values obtained in the selected models. It was calculated separately for the different algorithms.

The ME models were run with the feature classes L, Q, H, LQ and LQH (where L = linear, Q = quadratic, H = hinge), and a regularization multiplier (rm) from 0.5 to 2 by 0.5 steps. The selected method was also a k-fold strategy with five replicates. The area under the curve (AUC) and the AIC corrected for small sample sizes (AICc) were used to evaluate the models. Following these criteria, the model showing the lowest AICc was selected if the AUC value was above 0.8 [106]. For each hexaploid genetic-geographic group, the best-performing RF, ANN and GLM models (AUC-ROC > 0.95) and the best model resulting among ME models were selected to build an ensemble forecasting species distribution model. The ensemble forecasts are calculated as the mean of the individual model predictions weighted by AUC-ROC scores. To identify areas of probable ecological contact between pairs of hexaploid genetic-geographic groups, models were transformed to presence/absence. For this, cells with values over 0.5 were considered for presenting suitable habitats for the species.

This procedure is parallel to that followed by López-González et al. [41], where projections corresponding to distribution models onto the climatic scenarios of the present climatic conditions, as well as for those of the Mid-Holocene (6 ka BP) and Last Glacial Maximum (22 ka BP), were already published for the diploid species *V. dalmatica* in the study area.

#### 2.5.2. Niche Comparison Analyses

The 19 environmental layers of the BioClim dataset from the WorldClim database at 30 s (c. 1 km) resolution (Worldclim Version2; [107]), together with altitude information [108], were initially considered. Climatic niche comparisons were performed between pairs of ploidy levels (2*x* vs. 4*x*, 2*x* vs. 6*x* and 4*x* vs. 6*x*) given that it was our aim to explore niche differentiation among hexaploids and their putative ancestors. For niche quantification, it is recommended to filter records to avoid unequal representation of samples caused by sampling bias, which potentially would give rise to the assumption of independence among occurrence data [109]. Following this recommendation, a minimum distance filter was applied to prevent points closer than 10 km within each entity. All original records satisfied this criterion; thus, 50 presence records were considered for the analyses: 9 for the diploids, 9 for the tetraploids and 32 for the hexaploids. To elucidate the autocorrelation of variables, we calculated Pearson correlations. Within each set of variables showing a correlation <0.7, we selected those showing the lowest values of VIF. Eight bioclimatic variables were finally selected. For the niche comparison analysis, a multivariate analysis (PCA-env) was performed based on climatic data using the method developed by Broennimann et al. [110]. Niche overlap between hexaploids and its putative ancestors was calculated using the R package ‘ecospat’ [111]. This calculation is based on Schoener’s *D* metric [112] that ranges from 0 (no overlap) to 1 (complete overlap). The procedure and metrics are described in detail in Padilla-García et al. [113]. Niche breadth was also calculated following the procedure described in Theodoridis et al. [114], Kirchheimer et al. [115] and Padilla-García et al. [113]. The results were visualized using boxplots for each PCA axis.

## 3. Results

### 3.1. DNA Ploidy Level

Ploidy levels were newly estimated for 456 individuals according to the 1C-values obtained. Ninety-one percent of the measurements presented a sample coefficient of variation (CV) of G1 peaks below 5%, while the CV of the remaining measurements was between 5 and 7.9%. All values were accepted for ploidy level estimation as the CV was ≤10% [116]. Considering only measurements with CVs < 5%, genome size values ranged from 1.11 to 2.13 pg. The variation was not continuous, and the 1C-values were arranged in two groups corresponding to two different ploidy levels: 4*x* (populations 39, 45 and 47; Figure 1; Table S3) and 6*x* (remaining populations; see Figure 1; Table 1 and Table S3). Genome size values corresponding to the diploid level were not found (note that diploid populations included here were analyzed by flow cytometry in a previous study [41]). Nuclear DNA contents ranged between 1.11 and 1.32 pg for tetraploids and between 1.67 and 2.13 pg for hexaploids (Table S3). All populations were composed of a single cytotype.

### 3.2. Genetic Structure and Population Differentiation Based on SSR Markers

**Figure 2:**
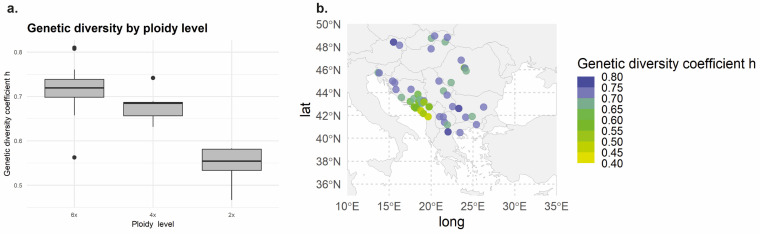
(**a**) Nuclear genetic diversity levels as compared across ploidy levels (*h:* Nei’s genetic diversity index). (**b**) Distribution of the estimated nuclear genetic diversity of the studied *Veronica* populations across the study area.

From the initial set of microsatellites, two were deprecated due to genotyping incongruence and the presence of indels. A total of 156 alleles were amplified from the 10 microsatellite loci that provided reliable genotypes. Genetic diversity patterns obtained using SPAGeDi (original matrix) were similar to those obtained using AFLPdiv and Popgene (presence/absence matrix). Thus, only the results derived from SPAGeDi are commented here. Table 1 shows the genetic diversity coefficient *h* (Nei’s genetic diversity index) as the most biologically meaningful summary statistic. The comparison of genetic diversity among ploidy levels (Figure 2a) and the spatial distribution of genetic diversity across the study area (Figure 2b) shows that genetic diversity values are highest in the hexaploid populations and lowest in the diploid ones.

**Figure 3:**
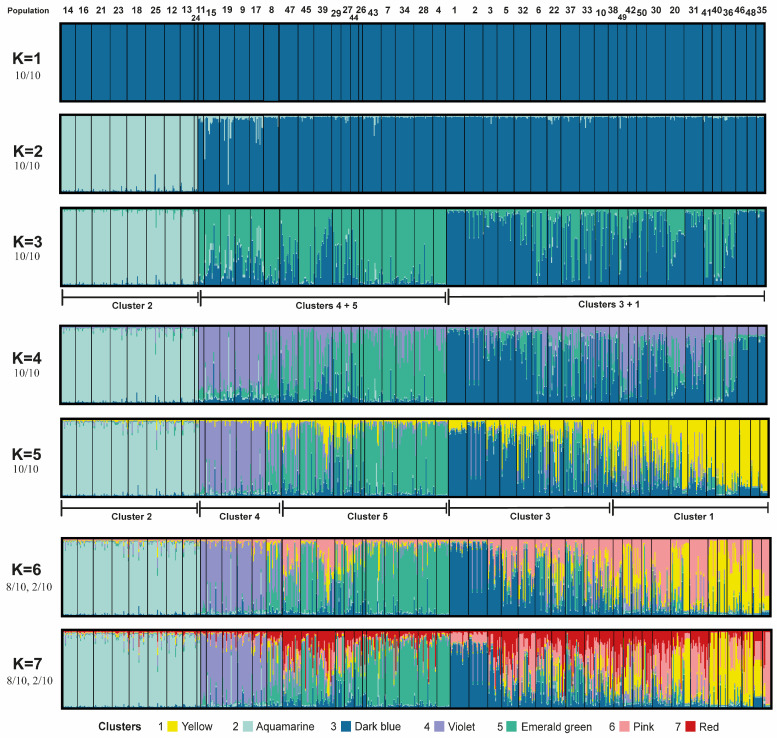
Bayesian clustering analyses (*K* = 1–7). Each vertical bar represents an individual, and the segments of each bar denote the probability of belonging to each inferred genetic cluster (represented by different colors). Brackets below the *K* = 3 and *K* = 5 plots identify the correspondence to specific population clusters. The fractions displayed under each *K* value (e.g., 10/10) represent the number of independent runs that reached the shown clustering solution.

According to the methods proposed by Evanno et al. [74] and Arrigo et al. [78] (for Structure and *K*-means, respectively), the best value for *K* is 2, with the next best values at *K* = 3 and then *K* = 5, although the differences in DeltaK values are very slight, as logically expected for an allopolyploid complex (Figure S1). Exploring higher values of *K* is justified, especially if population assignments make biological sense [117] and individuals are strongly assigned to all clusters [118]. Results derived from the Bayesian clustering analyses for *K* values from 1 to 7 are presented in Figure 3 for Structure and in Figure S2 for *K*-means, where the mostly concordant assignment of individuals to groups between both methods can be found (Figure S2).

Regarding the *K* = 3 clustering option derived from Structure (Figure 3), it is mostly congruent (ca. 70%) with the results obtained from non-hierarchical *K*-means (Figure S2), but only partially with DAPC (Figure S3). The identified genetic groups are: (1) the diploid individuals sampled; (2) a second genetic cluster composed of the tetraploids, plus the hexaploids extended to the north-eastern part of the region considered; and (3) a lineage composed only of hexaploids represented in the southern and in the north-western part of the Balkan Peninsula.

**Figure 4:**
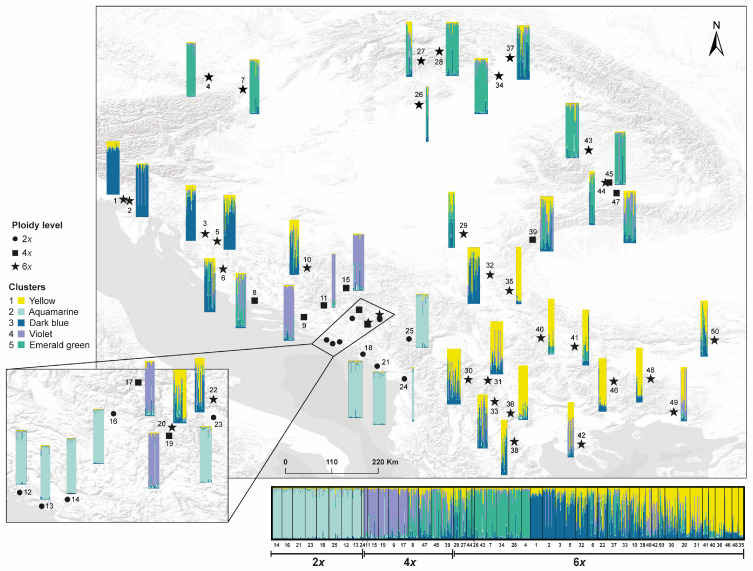
Spatial representation of the population genetic structure according to the Bayesian clustering (*K* = 5). The segments of each rectangle denote the probability of belonging to each cluster (i.e., SSR lineage). Clusters are defined by the following colors: (yellow) cluster 1-southern genetic-geographic group, hexaploids; (aquamarine) cluster 2-diploids; (dark blue) cluster 3-western genetic-geographic group, hexaploids; (violet) cluster 4-central-western tetraploids; and (emerald green) cluster 5-northern genetic-geographic group of hexaploids, plus three north-eastern tetraploid populations.

**Figure 5:**
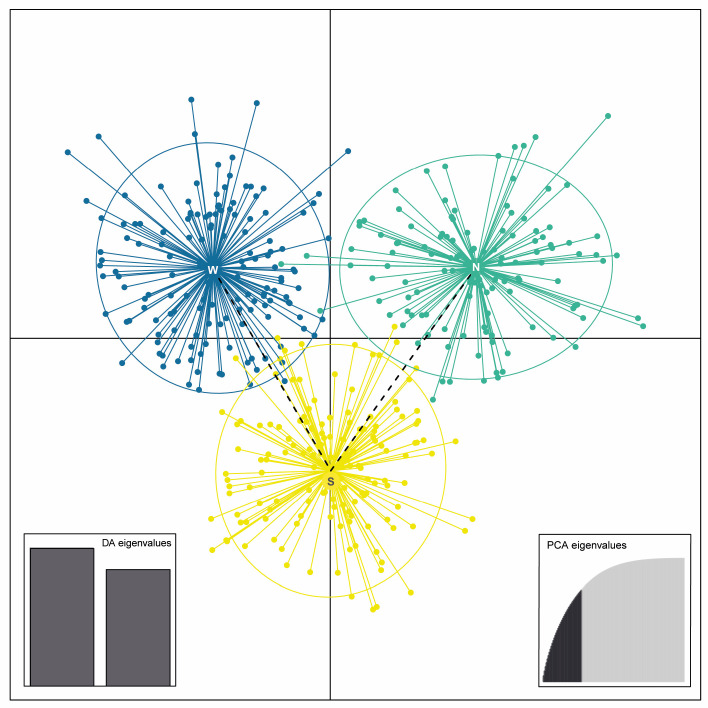
Scatterplot of Discriminant Analysis of Principal Components (DAPC) at *K* = 3 corresponding to the hexaploid species *V. austriaca*. Dots represent individuals. No *a priori* assignment of individuals to groups was applied. Clusters are defined by the following colors: (yellow) southern genetic-geographic group (S); (dark blue) western genetic-geographic group (W); and (emerald green) northern genetic-geographic group (N).

The general grouping found by Structure at *K* = 5 is overall consistent (again ca. 70%) with the results obtained from non-hierarchical *K*-means (Figure 3 and Figure S2, correspondingly) and DAPC (Figures S4 and S5; an interactive HTML version of this last figure, which allows visualizing different projections of the axes, is available in the supplementary material as FIGS5_3D_DAPC.html). This grouping option results in the following clusters (i.e., SSR lineages; Figure 4): one represented in the southern area that is conformed only by hexaploids (cluster 1, yellow) that is overall coincident with the southern haplogroup derived from plastid DNA sequence data (see below, Section 3.3.); a second lineage composed of diploids restricted to the south-east of the Neretva river (cluster 2, aquamarine); a third cluster, which is made of the hexaploids extended to the north-western part of the study area (cluster 3, dark blue); cluster 4 (violet) that is formed by the tetraploids from the central-western part of the region; and a fifth cluster (cluster 5, emerald green) mainly represented to the north (Austria, Hungary, Slovakia, and Romania), which is mostly composed of hexaploids, plus three geographically close tetraploid populations (pops. 39, 45 and 47). In this grouping option, clusters 2 (diploids) and 4 (tetraploids) result, therefore, mainly from differences in ploidy levels with respect to the remaining ones (mainly hexaploids, except for the three just mentioned populations). Although the Evanno method and the rate of change in likelihood might suggest *K* = 2 or *K* = 3 as more plausible solutions, the analysis at *K* = 5 prioritizes biological realism (consideration of ploidy levels), coherence among different data sources (congruence with plastid DNA sequence data in the recognition of a southern haplogroup) and among independent analyses, (bio-)geographic sense (see Discussion) and model stability. This decision is supported by the convergence analysis results and will therefore be discussed in detail onwards.

The next best grouping option identified by Structure (*K* = 4) can be observed in Figure 3, together with other suboptimal values of *K*.

The DAPC with no *a priori* assignment of individuals to groups as applied to the hexaploid species *V. austriaca* is shown in Figure 5. Three distinct genetic groups were found that fully correspond with the three genetic clusters that mostly contain hexaploids identified through the just described Bayesian analyses at *K* = 5 (i.e., clusters 1, 3 and 5). The superimposed minimum-spanning tree shows that the northern and western genetic groups are connected through the southern one.

Therefore, in order to facilitate discussion of results, three main genetic-geographic groups will be discussed onwards within the hexaploid species *V. austriaca*: (i) the southern group (cluster 1); (ii) the western group (cluster 3); and (iii) the northern group (cluster 5).

### 3.3. Analysis of the Plastid DNA Sequence Data

The total length of the 298 cpDNA sequences was 261–272 bp and 581–588 bp for the *trnH-psbA* and *ycf6-psbM* plastid regions, respectively. The two regions showed 13 (*trnH-psbA*) and 11 (*ycf6-psbM*) polymorphic sites, which led to a total of 37 haplotypes.

**Figure 6:**
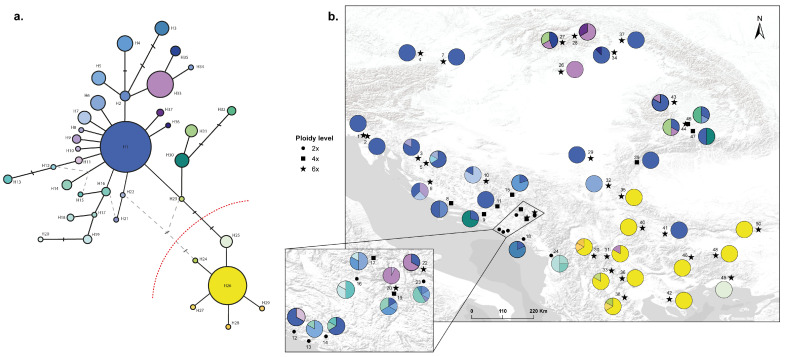
(**a**) Plastid haplotype network. Each color represents a different plastid haplotype. The dotted red line represents the division between two main haplogroups, while the discontinuous grey lines represent ambiguities (loops) that were resolved following Crandall and Templeton [87]. (**b**) Spatial representation of the plastid haplotypes with indication of ploidy levels (circles 2*x*; squares 4*x*; stars 6*x*) for each of the studied *Veronica* populations.

Overall, the resulting haplotype network was complex (Figure 6a), but two clear main haplogroups were found that are geographically well structured (Table 1; Figure 6b): a southern haplogroup, which corresponds with the southern genetic-geographic group and a northern haplogroup that included the remaining samples included in this study. Within the northern haplogroup, H1 was dominant, while H26 predominated within the southern haplogroup (Figure 6a), and both predominant haplotypes were connected by five parsimony mutation steps.

### 3.4. Morphometrics

Morphometric measurements comprised a total of 60 fruit and leaf characters, including those that were considered as quotients to avoid the size effect. Style and bract length, both measured in fruitful individuals (SL and BL), were the most helpful characters to distinguish individuals according to their ploidy level (Figure 6a,b). SL was significantly different between diploids and hexaploids, and BL discriminated significantly not only between that ploidy pair but also between hexaploids and tetraploids (Table 2).

Regarding specific differences between the cryptic *V. dalmatica* and *V. austriaca* ssp. *jacquinii*, the main morphometric characters used in the diagnosis of the first by Padilla-García et al. [40] were revisited. Style and bract length (SL and BL) were identified as the best diagnostic traits to distinguish between these two taxa (Figure S6; Table 3).

**Figure 7:**
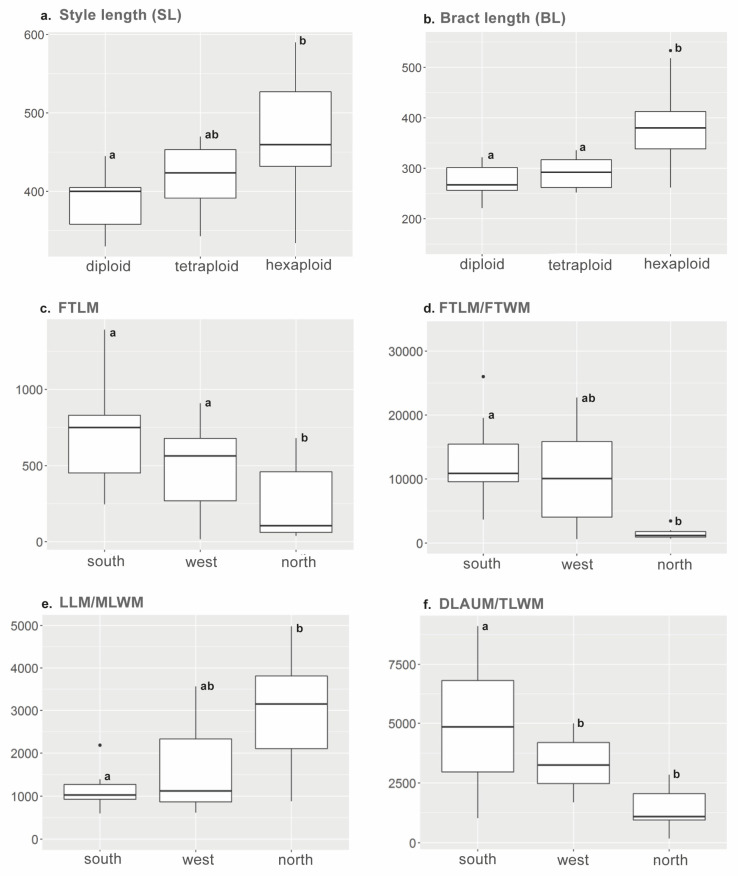
Boxplots representing the variation and median values of the most discriminant characters to distinguish among groups, according to different grouping options: (**a**) style length (SL) and (**b**) bract length (BL) variation among ploidy levels; (**c**–**f**) variation in the characters FTLM (the length of the first tooth of the mid-stem leaf), FTLM/FTWM (the ratio of the length to the width of the first tooth of the mid-stem leaf), LLM/MLWM (the ratio of the total length to maximum width of the mid-stem leaf) and DLAUM/TLWM (the ratio between the distance from the apex to the first tooth and the width of the entire terminal portion of this leaf) among the genetic-geographic groups identified within *V. austriaca*. Trait name abbreviations follow Table S2. Different letters indicate significant differences between groups (*p* < 0.05, Tukey’s HSD test).

When considering the three genetic-geographic groups obtained within the hexaploid *V. austriaca*, the most discriminant characters were related to leaf shape, which is in accordance with the traditional organization of the morphological variability of *V. austriaca* into three morphological groups or subspecies based on foliar traits. The most obvious differences appeared between the [southern genetic-geographic group + western one] *versus* [northern genetic-geographic group]. Thus, although the length of the first tooth of the mid-stem leaf (FTLM) showed slightly overlapping values between these two groups (Figure 7c), the differences were found to be significant (Table 4). The value of the ratio of the length to the width of the first tooth of the mid-stem leaf (FTLM/FTWM) was clearly higher in the southern genetic-geographic group as compared to the northern one (Figure 7d; Table 4), which indicates that the leaves of the mid-stem of the individuals from the southern genetic-geographic group are more divided. As for the individuals of the northern group, they have wider leaves compared to the other lineages, suggesting that the ratio of total length to maximum width of the mid-stem leaf (LLM/MLWM; Figure 7e; Table 4) could be used to distinguish between these genetic-geographic groups. Additionally, both the ratio maximum length-to-maximum width of the mid-stem leaf (LLM/MLWM) and the ratio between the distance from the apex to the first tooth and the width of the entire terminal portion of this leaf (DLAUM/TLWM; Figure 7f; Table 4) indicated a general tendency (more than a clearly significant differentiation in absolute values) to a morphological separation among the three groups.

### 3.5. Analyses Based on Environmental Variables

#### 3.5.1. Species Distribution Models for the Three Genetic-Geographic Groups Found Within *V. austriaca*

The following variables were finally selected: BIO8 (mean temperature of wettest quarter), BIO15 (precipitation seasonality), and BIO19 (precipitation of the coldest quarter) for the southern genetic-geographic group; BIO12 (annual precipitation), BIO15, and BIO18 (precipitation of warmest quarter) for the western genetic-geographic group; BIO19 and altitude for the northern one. From these, BIO8, BIO15 and BIO19 were the most important variables corresponding to the southern, western and northern groups, respectively. The variable contribution for each modelling approach is shown in Table 5. Although the values differed, the variables with the highest contribution for each genetic-geographic group were the same regardless of the algorithm employed.

**Figure 8:**
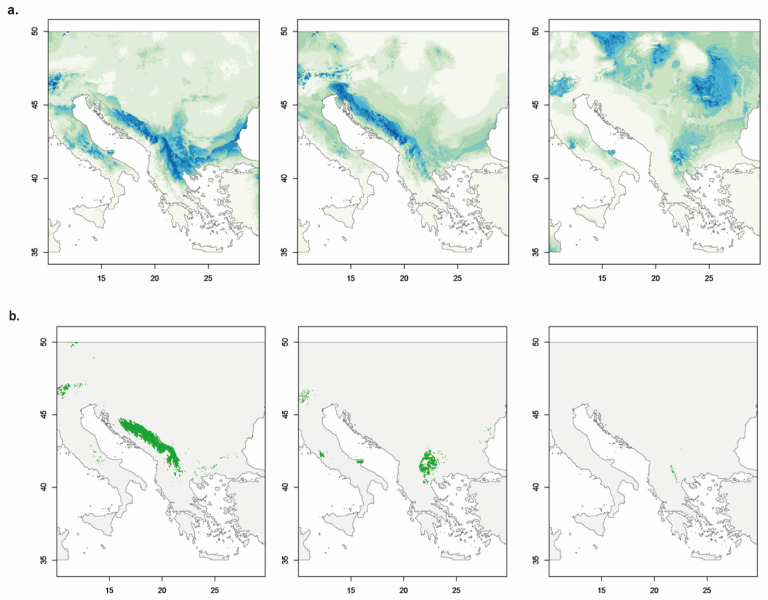
SDMs. (**a**) Potential suitability areas for the three main genetic-geographic groups found within the hexaploid species *V. austriaca*. From left to right: southern genetic-geographic group, western genetic-geographic group, and northern genetic-geographic group. Color gradient from white to green to blue indicates increasing habitat suitability. (**b**) Ecological contact zones between pairs of these genetic-geographic groups. From left to right: southern-western, southern-northern, and northern-western group contacts. Green areas indicate potential ecological contact zones between groups.

A total of 14 models were selected for the southern group, 15 for the western one and 9 for the northern lineage. No GLMs with AUC-ROC scores over 0.95 were found in the last case. The ME models selected were L for the first group with a regularization multiplier of 0.5; LQ for the second with the same regularization multiplier; and LQH with a regularization multiplier of 2 for the northern genetic-geographic group. These models were those showing the lowest AICc given that the lowest value of AUC was above the threshold established (0.85 for the northern lineage). The final ensemble forecasting models for each genetic-geographic group are shown in Figure 8a. The potential distribution of the southern lineage appears in the area south of the Danube throughout a wide range of altitudes, including the central and southern parts of the Dinaric Alps. The potential distribution area of the western genetic-geographic group extends throughout the Dinaric Alps and the western part of the Republic of North Macedonia. Potential suitable areas for this group also appear in the Alps. The potential distribution range of the northern genetic-geographic group extends from the south-eastern part of the Republic of North Macedonia, through the high areas of the Rhodope Mountains to the Carpathians (mainly south and western Carpathians) and reaches further areas to the north towards Central Europe.

Ecological contact zones among these three genetic-geographic groups (Figure 8b) were found between the southern-western lineages (in the central and southern parts of the Dinaric Alps and the western part of the Republic of North Macedonia) and between the southern-northern lineages (eastern part of the Republic of North Macedonia and western Bulgaria). Last, contact zones between the western and northern genetic-geographic groups were almost non-existent, except for a small corridor along the northern part of the Scardo-Pindic mountain system.

#### 3.5.2. Prediction of the Ecological Niche Optimum and Breadth for the Individuals from the Different Ploidy Levels

**Figure 9:**
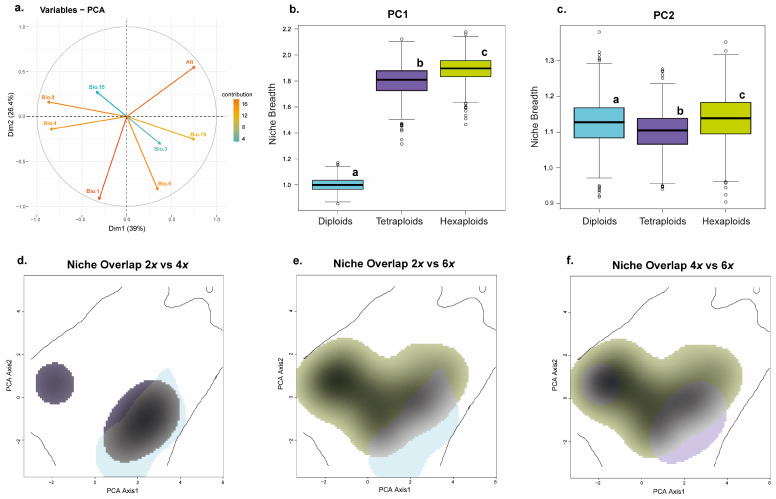
Climatic niche comparison analysis between 2*x* (turquoise), 4*x* (purple) and 6*x* (pistachio) individuals of the allopolyploid complex. (**a**) PCA-env and correlation circle of the eight environmental variables along the first two axes; (**b**,**c**) boxplots showing the niche breadth for 2*x*, 4*x* and 6*x* along the PC1 and PC2 axes; (**d**–**f**) Climatic niche comparisons of 2*x* vs. 4*x*, 2*x* vs. 6*x* and 4*x* vs. 6*x*, respectively. The fraction of the climatic niche that overlaps between different comparisons is shown in grey. The solid contour line indicates 100% of the available (background) environmental space.

The variation explained by the first two axes of the PCA-env was 39% and 26.4%, respectively, which means 65.4% of the total variance (Figure 9a). The contribution of each variable to the first two axes of the PCA is shown in Table S4 and Figure 9a. According to our analysis, hexaploids show a climatic niche significantly wider than that corresponding to their putative ancestors (Table S5; Figure 9d–f). Hexaploids show a low percentage of niche overlap with diploids (11%) and tetraploids (20%). The results also indicate a strong niche expansion of hexaploids with respect to diploids (E = 0.782) and tetraploids (E = 0.605). The niche unfilling index is also high with respect to diploids (U = 0.408), and the stability index is low (S = 0.218). In contrast, tetraploids have partially filled the niche occupied by hexaploids (U = 0.167, S = 0.395). A visual inspection of the boxplots reveals that along PC1, the niche breadth is higher in both tetraploids and hexaploids than in diploids and slightly higher in hexaploids compared with tetraploids (Figure 9b). Along PC2, no substantial differences in niche breadth are found among ploidies (Figure 9c).

## 4. Discussion

### 4.1. Genetic, Ecological and Morphological Variability of Veronica austriaca and Its Relatives

Allopolyploids incorporate genetic variability from multiple progenitor populations, leading to increased nuclear genetic diversity [119]. According to the expectations, the hexaploid *V. austriaca* displays higher levels of genetic diversity than the diploid (*V. dalmatica*) involved in its formation (Table 1; Figure 2). This is congruent with the broader distribution of the hexaploid. Indeed, a spatial representation of the estimated genetic diversity displayed by the populations belonging to the allopolyploid complex across the study area (Figure 2b) shows that the lowest levels of genetic diversity in the group are concentrated in the central-western part of the Balkan Peninsula, where the lower ploidy levels 2*x* and 4*x* are mainly represented. Additionally, the genetic diversity values are maximum in the hexaploid populations and minimum in the diploid ones (Figure 2a). Increased intraspecific nuclear genetic diversity associated with allopolyploidization can lead to increased fitness, adaptability and competitiveness [120], which in this case would correspond to the hexaploid species. A higher level of genetic diversity of the hexaploid combined with an overall moderate genetic differentiation among the three genetic-geographic groups detected within it (i.e., admixture levels in Structure analysis, Figure 4, as well as results derived from DAPC, Figure 5) suggest in this case the existence of inter-population and inter-lineage gene flow. Although it is not a guarantee, maintaining genetic diversity within natural populations is crucial to maximizing the potential of a species to survive in a changing environment [121], which is important in the allopolyploid complex studied here that is representative of mesic meadows and other Eurasian grassland areas.

Genetic isolation seems to be higher among ploidy levels than among the three clusters detected within the hexaploid *V. austriaca* (Figure 4), particularly between the diploids and the tetraploids distributed in the western part of the Balkan Peninsula. Additionally, the populations of the diploid *V. dalmatica* and the uncertain tetraploid display the lowest levels of genetic diversity and are usually composed of a low number of individuals. Consequently, in case connectivity is lost (e.g., habitat fragmentation), genetic drift could negatively affect these small, isolated populations, influencing their genetic structure and increasing among-population differentiation [122]. Thus, at least the populations of the endemic *V. dalmatica* would deserve protection measures.

The hexaploid species *V. austriaca*—which is more widely distributed than its lower ploidy ancestors, particularly to the north and east of the Balkan Peninsula (Figure 1)—shows relatively high tolerance to different environmental conditions according to its niche expansion and niche breadth observed in our analyses (Table S5; Figure 9). Hence, its potential distribution area covers a great extent of the Balkan Peninsula (Figure 8) and extends northwards to the Central European mixed forests ([39]; according to Borissova [43], it is also present in Belarus, European Russia, Moldavia, Ukraine and S Caucasus). Considering only the Balkan Peninsula and the parts of Central Europe surveyed here, and according to the Digital Map of European Ecological regions (DMEER; [123]) the 6*x* populations of *V. austriaca* are found across a minimum of ten ecologically distinct areas (including six types of mixed forests—Balkan, Rhodope montane, Pindus mountains, Dinaric mountains, Pannonian and Central European mixed forest—the Aegean sclerophyllous forest, the Illyrian deciduous forest, the Carpathian montane coniferous forest and the East European forest steppe), while the diploid and tetraploid representatives of the group are confined to just three of them. Rojas-Andrés et al. [124] have proposed that the associated changes related to polyploidization and genome downsizing might have contributed to the colonization of new habitats by the species of *V*. subsect. *Pentasepalae*, at least at a broad scale. Decomposing single species into genetic units may represent a powerful approach to better understanding their distribution ranges [35]. The ecological tolerance of the hexaploid *V. austriaca* is probably the result of the sum of the different requirements of the three molecular lineages identified within the species (Figure 8) that are nowadays distributed across different ecological zones identified in the area.

In addition to its genetic and ecological variability, *V. austriaca* shows phenotypic variability of leaf characters. Specifically, leaves are larger and less divided in the individuals of the northern genetic-geographic group as compared with those of the individuals from the southern and western lineages (Figure 7). This is consistent with the traditional taxonomic treatment of *V. austriaca* into three morphological groups or subspecies based on foliar characters (e.g., [41,44]). While the southern and the western genetic-geographic groups correspond, in general terms, with *V. austriaca* subsp. *jacquinii*, the northern group mostly encompasses the other two subspecies (i.e., *V. austriaca* subsp. *austriaca* and *V. austriaca* subsp. *dentata*). Larger leaves lead to increased vegetative biomass and are considered adaptive in wet environments [125,126]. Likewise, one of the most noticeable effects in plant adaptation to water deficit is the leaf area reduction (i.e., the evaporative surface) [127]. Recent studies focused on genetic intraspecific units within wide-range species have demonstrated local adaptation by modifying traits and adjusting physiological features to different environmental conditions [128,129]. Altogether, the present results suggest a possible adaptive value of the leaf characters studied in *V. austriaca*, but further studies focused on these particular issues are needed to demonstrate it.

Moreover, increased morphological variance, as observed in the studied characters, may just derive from phenotypic plasticity, but it can also be genetically based and the result of evolutionary responses to ecological opportunity. Phenotypic variability may facilitate colonization of new environments by shifting the population mean phenotype in a way that increases niche availability [130]. The success of *V. austriaca* (i.e., niche expansion and more extensive niche breadth in its distribution area, as well as a higher capacity to occur in different grassy habitats and ecological units across its distribution range, as compared to those of its lower ploidy ancestors) could be the result of the great genetic and morphological diversity that would have allowed this species to respond to a wide range of environmental conditions.

Last, the phenotypic differences between *V. austriaca* ssp. *jacquinii* and the recently described *V. dalmatica* were here revisited. *Veronica dalmatica* (2*x*) and *V. austriaca* ssp. *jacquinii* (6*x*) are a pair of morphologically very close taxa (i.e., the first one is considered a cryptic species that was traditionally included within the variation of the second). Style and bract length (SL and BL) were identified as the best diagnostic traits to distinguish between these two taxa (Figure S6), with the diploid *V. dalmatica* showing significantly shorter styles than the hexaploid *V. austriaca* ssp. *jacquinii*. BL was not originally explored [40], but our results demonstrate its utility for taxon identification. A larger bract may provide additional protection of flower buds against late frosts and thus ensure that some flowers of the raceme overcome this problem. The potential impact of these characters related to pollination differences should be explored, as well as possible differences in pollen size related to ploidy level, given that such associations have been previously described in the genus and even within this section of *Veronica* [131,132]. Style length and corolla diameter (which wasnot explored in this study) could be inversely related to selfing rates. There is a well-known strong correlation between pollen/ovule (P/O) ratios and breeding systems: P/O ratios decrease from xenogamous to facultative xenogamous to autogamous species. Current data show that larger pollen grains are associated with lower pollen counts and higher selfing rates [133]. However, the breeding systems of *Veronica* are not well understood, and there is a significant lack of data regarding their pollen–ovule ratios among other parameters. To fill this gap, more in-depth studies are required to investigate these characteristics and how they relate to ploidy and breeding systems.

### 4.2. Phylogeography of V. austriaca

#### 4.2.1. Putative Origin and Expansion of *V. austriaca*

It has recently been proposed that the Austrian speedwell allopolyploid complex geographically originated in the Western Balkans, specifically in the southern half of the Dinaric Alps [41]. Based on the available evidence (e.g., suggested presence of the putative parents in the same area at that time [41]), *V. austriaca* would have had its origin during the post-glacial period. From this area, *V. austriaca* would have spread mainly in three directions: to the south-east (Balkan-Aegean area), to the northwest (Dinaric-Illyrian area), and to the northeast (Pannonian-Carpathian area). This hypothesis is congruent with both the three molecular lineages found within the hexaploid and with the genetic distances observed among these lineages (i.e., the northern and western genetic-geographic groups are genetically connected through the southern one according to the minimum-spanning tree superimposed to the DAPC; Figure 5), but would probably need further specific testing using genomic methods.

The south-eastward expansion of the group is represented by the southern genetic-geographic group (Figure 4), which is mostly coincident with the southern haplogroup (Figure 6). This lineage most likely reached the Balkan-Aegean area through the northern part of the Scardo-Pindic mountain system (in the north-western part of the Republic of North Macedonia [134]), which represents a geographic continuation of the southern Dinaric Alps. The ecological contact area between the southern and western genetic-geographic groups (Figure 8b) matches with this “corridor”, which may have facilitated colonization and genetic exchange. This idea is also supported by the presence of haplotypes that connect the two main haplogroups (i.e., northern and southern) along this area (e.g., H16 in population 24, H23 in population 33, etc.; Figure 6). Also, the populations that occur in this area (30, 31, 33 and 38) show high levels of admixture (Figure 4), which suggests that this area may represent a “transitional zone” among lineages.

Furthermore, a potential connection has been detected between the southern and northern genetic-geographic groups via the Carpathian-Balkan mountain ranges. The presence of an haplotype that belongs to the northern genetic-geographic group in western Bulgaria (H1 in pop. 41, see Figure 6) could suggest recent north to south gene flow. Since cpDNA is maternally inherited (via seeds), this would indicate that the northern populations acted as “mothers”, implying seed transport—a dispersal mechanism generally less efficient than pollen, and particularly less sophisticated in *Veronica* (seeds without obvious structures for long-distance dispersal). Alternatively, the presence of this haplotype could be interpreted as a ‘relict haplotype’ belonging to the western genetic-geographic group, as this lineage could possibly have had a wider distribution in the southern area but was subsequently displaced by the current southern lineage and haplotype group. Although these two regions (north and south) have distinct floras and faunas, the existence of this isolated haplotype suggests some levels of genetic exchange between them [6]. However, given the singularity of this finding, more exhaustive sampling in that area would be essential to confirm the nature and extent of this possible connection.

The northern expansion of *V. austriaca* is best represented by haplotype H33 (Figure 6), which connects the hypothetical original area of *V. austriaca* in the southern half of the Dinaric Alps (e.g., pops. 20 and 22) with more northern populations (pops. 26, 27, 28, 43, and 44). This lineage corresponds to the SSR northern genetic-geographic group (cluster 5, Figure 4). The populations of this northern group are distributed throughout northern Austria, Slovakia, northern Hungary, and Romania. The north-eastern dispersal seems to have reached Serbia and southern Romania (pops. 29, 39, 44; Figure 4 and Figure 6) via the natural gorge of the “Iron Gates” as a possible corridor between the Dinaric Alps and the Carpathians, as has been previously described for other species [135,136]. These latter populations within the northern lineage are located mainly in the Transylvanian Basin (between the Apuseni Mountains and the eastern part of the Carpathians), as well as in the north-western part of the Carpathians. While many species from the Carpathian Mountains show their genetic variation structured in two groups—i.e., north-western Carpathian group *versus* south-eastern Carpathian one [e.g., Mráz et al. [137] with *Hypochaeris uniflora* Vill. and Ronikier et al. [14] with *Campanula alpina* Jacq.]—, the northern populations of *V. austriaca* studied here are genetically homogeneous (Figure 4). The genetic variation structured in two groups found in many Carpathian species has been proposed to be the consequence of habitat dynamics during the Quaternary climatic oscillations [138]. In contrast, the genetic uniformity found in the northern lineage could be interpreted as evidence of a gradual post-glacial expansion following Quaternary climatic oscillations, rather than prolonged isolation. In fact, some authors have suggested that the Apuseni Mountains were not severely affected by the last glaciation and harboured scattered refugial stands situated at low and mid elevations during the early Holocene [139]. These areas would have acted as corridors that facilitated northern migrations and maintained gene flow [140].

The north-western expansion of *V. austriaca* is represented by the western genetic-geographic group, which consists of populations that extend along the north of the Dinaric Alps (e.g., pops. 1–6). This expansion is similar to one of the possible post-glacial recolonization routes found for *Carpinus betulus* L. [141]. Data from nuclear markers suggest a pattern of increasing introgression in a southerly direction (Figure 4). To the south of this mountain range, particularly in Bosnia and Herzegovina and Montenegro (pops. 20 and 22), the hexaploid individuals are genetically intermediate between the western genetic-geographic group and the southern one. In contrast, the populations located to the north belonging to this lineage (pops. 1, 2, 3, and 5) exhibit remarkable genetic homogeneity (Figure 4). This pattern of post-glacial expansion has also been observed in genera such as *Edraianthus* A. DC. or *Heliosperma* (Rchb.) Rchb. [136,142] in the territories of Italy, Croatia, and Slovenia.

#### 4.2.2. The Balkan Peninsula as a Crossroad of Lineages

The southern Balkan Peninsula has been identified as one of the areas with the highest concentration of endemic species in Europe [143,144]. Among the regions that are notable for their diversity, the Dinaric Alps are considered a relevant speciation hotspot [145], while both the Carpathians and the Pannonian Plains represent key biodiversity foci for multiple taxa [146,147]. The three different lineages present within *V. austriaca* seem to be the result of geographical diversification in these areas. Other species of the genus *Veronica* show comparable genetic patterns with higher differentiation among lineages due to even more complex biogeographical scenarios dating from the mid-Pleistocene [148,149].

As is the case with several species native to the Mediterranean (e.g., [140,150,151], the present distribution of *V. austriaca* can be explained by its spread from the southern Dinaric Alps due to post-glacial expansion during the Holocene.

Furthermore, the results obtained suggest a complex introgression pattern along its dispersal routes. Consistent with what has been stated, this is observed through high admixture levels in populations in the south-eastern part of Bosnia and Herzegovina, northern Montenegro, southern Serbia, and parts of North Macedonia (e.g., pops. 20, 22, 30–32) (Figure 4), an area that also shows high levels of haplotype mixing (e.g., pops. 16, 17, 19, 23) (Figure 6). Therefore, these populations are located in an area that represents a biogeographical intersection among the south-eastern Dinaric Alps (western genetic-geographic group), the Carpathians (that connect with the northern genetic-geographic group), and the Balkan Mountains, Pindus Range, and Rhodope Mountains (that link with the southern genetic-geographic group).

### 4.3. On the Importance of Allopolyploidy in the Colonization Abilities and Evolution of V. austriaca

The current analyses of the present-day diversity, population structure and biogeography of the Austrian speedwell species complex provide general insights on the colonization of an allopolyploid perennial herb across a large geographic area and a wide spectrum of mesic grassy habitats. It has been inferred that the colonization by *V. austriaca* occurred with moderate gene flow among populations and most probably started from the southwestern populations followed by consecutive colonization events in several directions. The colonizing ability of *V. austriaca* could have relied on its allopolyploid nature. Although the relationship between allopolyploidization and expansion abilities is species-specific, there are examples of allopolyploids that exhibit broader ecological and geographic ranges than those of their diploid relatives, which suggest a potential role of hybridization in niche expansion (e.g., [23,152]). Increased intraspecific genetic diversity associated with allopolyploidization can lead to increased fitness, adaptability and competitiveness [120]. Allopolyploidy can also be responsible for increased phenotypic variation [153,154], which could in turn increase environmental tolerance.

Along with the increased genetic diversity and its consequences, the recurrent formation of polyploids is also a widespread phenomenon, which has already been demonstrated for *V.* subsect. *Pentasepalae* [40]. Multiple origins of allopolyploids may be responsible for their ecological success by generating variability in the ecological traits underlying local adaptation and niche expansion [155]. Particularly within the Austrian speedwell allopolyploid complex, the geographic break-line reflected by the two main haplogroups (Figure 6) could be the result of (at least) two different allopolyploid origins/events in the southern part of the Dinaric Alps or in other areas. In this latter case, the tetraploids from the southern part of the Pannonian Plains-Carpathian range area may have acted as progenitors in the formation of the northern genetic-geographic group and haplogroup (Figure 3 and Figure 6). According to the paleodistribution models available for *V. dalmatica*—the (until now) unique diploid parental identified of *V. austriaca*—there were ecologically suitable areas for this plant in the mentioned area during the LGM [41]. Nevertheless, the presence of this species in the area does not seem probable due to the distance from the main distribution area of *V. dalmatica* (Dalmatian region) and the absence of intermediate locations with available adequate habitats that could have favoured the migration of the species. Maybe other alternative diploid parental species showing similar ecological requirements could have been involved in the hypothetical allopolyploidization event. Niche expansion in this case would be the product of multiple origins in which either the same or different parental species would have been implicated. Niche overlap was found to be relatively high for the pair diploid-tetraploid (Table S5; Figure 9d), which would further favour the idea that these ploidy levels could have spatially coincided in a wider array of places. Hypothetic secondary contacts among haplogroups would have decreased the genetic distance among populations, thus generating the current moderate genetic differentiation.

## 5. Conclusions

The hexaploid species *Veronica austriaca* displays high nuclear genetic diversity, ecological tolerances and morphological variability. Compared with their lower ploidy relatives, it shows increased size and extends its ecological niche and distribution range further beyond them. The range expansion of *V. austriaca* across the Balkan Peninsula and further on to colonize grassland areas of Eurasia may thus be explained by one or several ecologically successful allopolyploid events. It is possible that one allopolyploidization event led to increased ecological amplitude resulting in rapid colonization of new locations. Nevertheless, it cannot be ruled out that multiple allopolyploidization events may have promoted range expansion by generating different outcomes displaying distinct ecological tolerances. These hypotheses need to be revisited as they obviously demand further testing. Although the present results point in that direction, whether allopolyploidy is responsible for the colonizing ability of *V. austriaca* requires additional experimental studies on comparative fitness.

## Figures and Tables

**Table 1:** Summary characteristics per population describing ploidy level, assignment to one of the five clusters detected using microsatellites, haplotypes present in the population, assignment to one of the two haplogroups found, sample size and genetic diversity assessed through microsatellites (*h*: Nei’s genetic diversity index). Sample size refers to the number of individuals analyzed in each population.

Population	Ploidy	Assignment to Clusters as Detected by SSRs	Haplotype	Haplogroup	Sample Size	*h*
Pop. 1	6*x*	Cluster 3	H1	Northern	20	0.6885
Pop. 2	6*x*	Cluster 3	H1	Northern	20	0.7066
Pop. 3	6*x*	Cluster 3	H1 and H8	Northern	15	0.7452
Pop. 4	6*x*	Cluster 5	H1	Northern	14	0.7604
Pop. 5	6*x*	Cluster 3	H1, H12 and H22	Northern	18	0.7205
Pop. 6	6*x*	Cluster 3	H1, H7 and H9	Northern	17	0.7017
Pop. 7	6*x*	Cluster 5	H1	Northern	15	0.7494
Pop. 8	4*x*	Cluster 4	H1 and H2	Northern	16	0.6889
Pop. 9	4*x*	Cluster 4	H1 and H30	Northern	16	0.6314
Pop. 10	6*x*	Cluster 3	H1 and H7	Northern	15	0.7062
Pop. 11	4*x*	Cluster 4	H1	Northern	6	0.6563
Pop. 12	2*x*	Cluster 2	H1 and H11	Northern	17	0.5828
Pop. 13	2*x*	Cluster 2	H5 and H14	Northern	15	0.5815
Pop. 14	2*x*	Cluster 2	H1, H14 and H15	Northern	15	0.5811
Pop. 15	4*x*	Cluster 4	H1 and H4	Northern	17	0.6500
Pop. 16	2*x*	Cluster 2	H13, H19 and H20	Northern	17	0.5542
Pop. 17	4*x*	Cluster 4	H4, H6 and H19	Northern	15	0.6865
Pop. 18	2*x*	Cluster 2	H1 and H3	Northern	20	0.4723
Pop. 19	4*x*	Cluster 4	H1, H4, H5 and H14	Northern	16	0.6846
Pop. 20	6*x*	Cluster 1	H10 and H33	Northern	20	0.6574
Pop. 21	2*x*	Cluster 2	-	-	20	0.5335
Pop. 22	6*x*	Cluster 3	H1 and H33	Northern	15	0.7380
Pop. 23	2*x*	Cluster 2	H2, H3, H5, H16 and H21	Northern	18	0.5428
Pop. 24	2*x*	Cluster 2	H16, H17 and H18	Northern	4	0.4668
Pop. 25	2*x*	Cluster 2	-	-	20	0.5571
Pop. 26	6*x*	Cluster 5	H33	Northern	4	0.7374
Pop. 27	6*x*	Cluster 5	H31, H33 and H35	Northern	10	0.6749
Pop. 28	6*x*	Cluster 5	H33 and H37	Northern	20	0.7234
Pop. 29	6*x*	Cluster 5	H1	Northern	10	0.7140
Pop. 30	6*x*	Cluster 1	H26, H28 and H29	Southern	20	0.7453
Pop. 31	6*x*	Cluster 1	H26 and H33	Southern	20	0.7384
Pop. 32	6*x*	Cluster 3	H6	Northern	18	0.6795
Pop. 33	6*x*	Cluster 3	H23 and H26	Southern	16	0.7186
Pop. 34	6*x*	Cluster 5	H1 and H36	Northern	20	0.6654
Pop. 35	6*x*	Cluster 1	H26	Southern	8	0.7135
Pop. 36	6*x*	Cluster 1	H26	Southern	15	0.6705
Pop. 37	6*x*	Cluster 3	H1	Northern	20	0.7168
Pop. 38	6*x*	Cluster 1	H24, H26 and H27	Southern	10	0.8098
Pop. 39	4*x*	Cluster 5	H1	Northern	20	0.6871
Pop. 40	6*x*	Cluster 1	H26	Southern	10	0.7087
Pop. 41	6*x*	Cluster 1	H1	Southern	10	0.8078
Pop. 42	6*x*	Cluster 1	H26	Southern	10	0.7384
Pop. 43	6*x*	Cluster 5	H1 and H33	Northern	20	0.7309
Pop. 44	6*x*	Cluster 5	H1, H31 and H33	Northern	9	0.5626
Pop. 45	4*x*	Cluster 5	H1, H32 and H34	Northern	17	0.7417
Pop. 46	6*x*	Cluster 1	H26	Southern	12	0.7333
Pop. 47	4*x*	Cluster 5	H1 and H30	Northern	20	0.6595
Pop. 48	6*x*	Cluster 1	H26	Southern	10	0.6628
Pop. 49	6*x*	Cluster 1	H25	Southern	10	0.7499
Pop. 50	6*x*	Cluster 1	H26	Southern	11	0.7306

The symbol “-” indicates missing data.

**Table 2:** Results of the ANOVA for the comparison of style length (SL) and bract length (BL) between ploidy levels. The table shows the Levene’s Test *p*-value, the F-statistics, and the Tukey HSD test *p*-value for the comparisons between ploidy levels (last three columns).

Character	Homogeneity Test (Levene’s *p*)	F	*p* (ANOVA)	Post Hoc Test	2*x*–4*x*	2*x*–6*x*	4*x*–6*x*
SL	0.2029	6.4	** ^1^	Tukey HSD	0.7005	0.006	0.0959
BL	.	17.7	*** ^1^	Tukey HSD	0.7730	0.00001	0.0005

^1^ Asterisks indicate significance levels: *p* < 0.001 ‘***’, *p* < 0.01 ‘**’, *p* < 0.1 ‘.’.

**Table 3:** Results of the ANOVA for the comparison of the style length (SL) and bract length (BL) between *V. austriaca* ssp. *jacquinii* and the cryptic *V. dalmatica*. The table shows the Levene’s Test *p*-value, the F-statistics, and the Tukey HSD test *p*-value for the comparison between *V. austriaca* ssp. *jacquinii* and *V. dalmatica* (last column).

Character	Homogeneity Test (Levene’s *p*)	F	*p* (ANOVA)	Post Hoc Test	*V. austriaca* ssp. *jacquinii* vs. *V. dalmatica*
SL	0.246	7.6	** ^1^	Tukey HSD	0.0084
BL	0.058	11.3	** ^1^	Tukey HSD	0.0014

^1^ Asterisks indicate significance levels: *p* < 0.01 ‘**’.

**Table 4:** Results of the ANOVA for the comparison of the length of the first tooth of the mid-stem leaf (FTLM), the ratio of the length to the width of the first tooth of the mid-stem leaf (FTLM/FTWM), the ratio of total length to maximum width of the mid-stem leaf (LLM/MLWM) and the ratio between the distance from the apex to the first tooth and the width of the entire terminal portion of this leaf (DLAUM/TLWM) among genetic-geographic groups. The table shows the Levene’s Test *p*-value, the F-statistics, and the Tukey HSD test *p*-value for the comparison among genetic-geographic groups: Southern (S), Northern (N), and Western (W) (last three columns).

Character	Homogeneity Test (Levene’s *p*)	F	*p* (ANOVA)	Post Hoc Test	S—N	W—N	W—S
FTLM	0.4095	5.2	* ^1^	Tukey HSD	0.0459	0.0134	0.7640
FTLM/FTWM	0.2754	3.6	* ^1^	Tukey HSD	0.0391	0.4516	0.2875
LLM/MLWM	0.865	4.05	* ^1^	Tukey HSD	0.0295	0.0874	0.8524
DLAUM/TLWM	0.1838	12.9	*** ^1^	Tukey HSD	0.0003	0.3777	0.0062

^1^ Asterisks indicate significance levels: *p* < 0.001 ‘***’, *p* < 0.05 ‘*’.

**Table 5:** Variable contribution for the different algorithms employed in SDMs. The variable with the highest contribution in each case appears in bold and in a dark grey background. BIO8: mean temperature of wettest quarter; BIO12: annual precipitation; BIO15: precipitation seasonality; BIO19: precipitation of coldest quarter. RF: random forest; ANNs: artificial neural networks; GLMs: generalized linear models.

Algorithms	MaxEnt			RF			ANNs			GLMs		
Lineages *	South	West	North	South	West	North	South	West	North	South	West	North
BIO8	**50.8**	8.69		**43.90**	24.07		**40.87**	25.35		**48.24**	8.29	
BIO15	35.45	**85.9**		33.23	**43.76**		30	**43.75**		32.59	**90.95**	
BIO19	13.75		**85.7**	22.86		**58.9**	29.13		**63.7**	19.16		-
BIO12		5.41			32.16			30.89			0.76	
Altitude			14.3			41.1			36.3			-

* Genetic-geographic groups (lineages) based on Bayesian clustering of SSRs. (-) There were no models fitting the AUC criteria.

## Data Availability

The data supporting the findings of this study are available within the article and its Supplementary Materials (Minimal Dataset). Genetic sequences have been deposited in the European Nucleotide Archive (ENA) and will be made public upon publication.

## Supplementary Materials

The following supporting information can be downloaded at: https://www.mdpi.com/article/10.3390/plants15060955/s1.

## Abbreviations

The following abbreviations are used in this manuscript:

SSR	Simple Sequence Repeat
FCM	Flow Cytometry
cpDNA	Chloroplast DNA
dNTP	Deoxynucleotide Triphosphate
MCMC	Markov Chain Monte Carlo
DAPC	Discriminant Analysis of Principal Components
GLM	Generalized Linear Model
RF	Random Forest
ANN	Artificial Neural Network
ME	Maximum Entropy
AUC-ROC	Area Under the Receiver Operating Characteristic Curve
AICc	Akaike Information Criterion corrected
P/O	Pollen/Ovule ratio
LGM	Last Glacial Maximum
