# Supplementary material for: From the Balkan Peninsula to the Mesic Grassland Areas of Central Europe: Morpho-Genetic Diversity and Niche Differentiation in the Allopolyploid Complex of the Austrian Speedwell
Source: Plants (Basel). 2026 Mar 20;15(6):955. doi: 10.3390/plants15060955 (PMC13030098; doi:10.3390/plants15060955)
Supplement: Supplementary file 1 [file plants-15-00955-s001.zip › Supplementary material_V.austriaca.pdf]

Supplementary Data

From the Balkan Peninsula to the mesic grassland areas of Central Europe: Morpho-genetic diversity and niche differentiation in the allopolyploid complex of the Austrian speedwell.

David Jiménez-García[a,b,*], Noemí López-González[a,b], Daniel Pinto-Carrasco[a,b], Nélida Padilla-García[a,b], Santiago Andrés-Sánchez[a,b], Blanca M. Rojas-Andrés[a,b], M. Montserrat Martínez-Ortega[a,b,*]

[a] *Área de Botánica, Universidad de Salamanca, 37007 Salamanca, Spain*

[b] *Herbario y Biobanco de ADN Vegetal, Universidad de Salamanca, 37007 Salamanca, Spain*

*Corresponding authors.

E-mail addresses: david.jimgar@usal.es (D. Jiménez-García), mmo@usal.es (M. M. Martínez-Ortega).

The following Supporting Information is available for this article:

Fig. S1. DeltaK values for STRUCTURE.

Fig. S2. K-means clustering analysis (K = 1–7).

Fig. S3. Scatterplot of Discriminant Analysis of Principal Components (DAPC) at K=3.

Fig. S4. Scatterplot of Discriminant Analysis of Principal Components (DAPC) at K=5.

Fig. S5. 3D representation of the Discriminant Analysis of Principal Components (DAPC) for K = 5.

Fig. S6. Boxplots representing the variation and median values of the most discriminant characters to distinguish between *V. dalmatica* and *V. austriaca* ssp. *jacquinii*.

Table S1. Voucher information of the populations of *Veronica* included in this study.

Table S2. Leaf and fruit characters measured and their abbreviations.

Table S3. Newly generated genome size estimations and ploidy level inferences (1Cx, 1C and 2C-values), internal standards, sample and standard CV for the individuals included in the present study.

Table S4. Contribution of environmental variables to the first two principal components (PC1 and PC2) of the PCA-env.

Table S5. Niche overlap percentage and climatic niche dynamics (expansion, stability and unfilling) for each comparison.

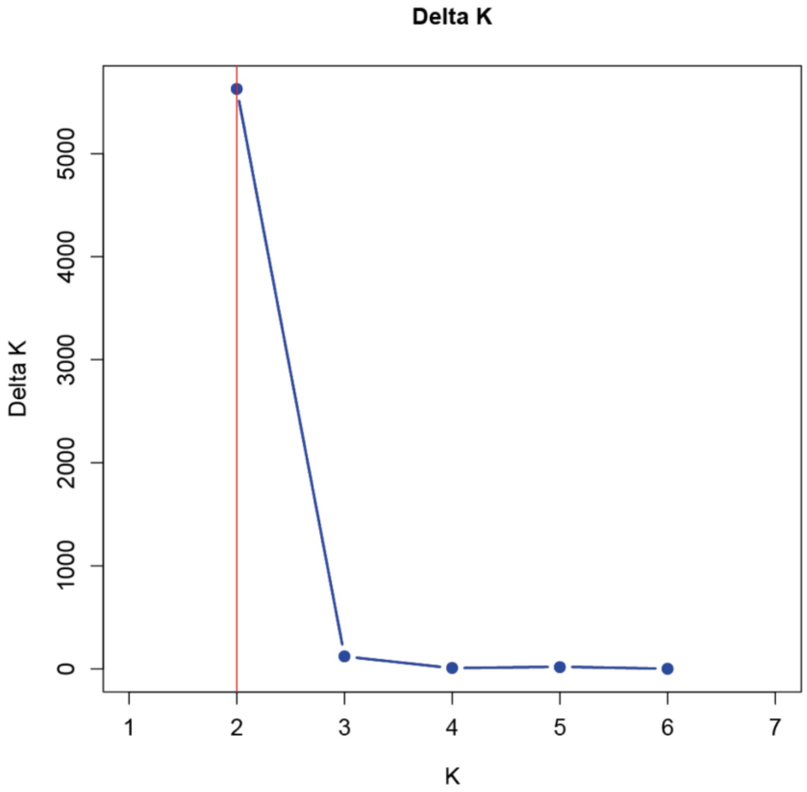

Fig. S1. DeltaK values for STRUCTURE according to the method proposed by Evanno et al. (2005).

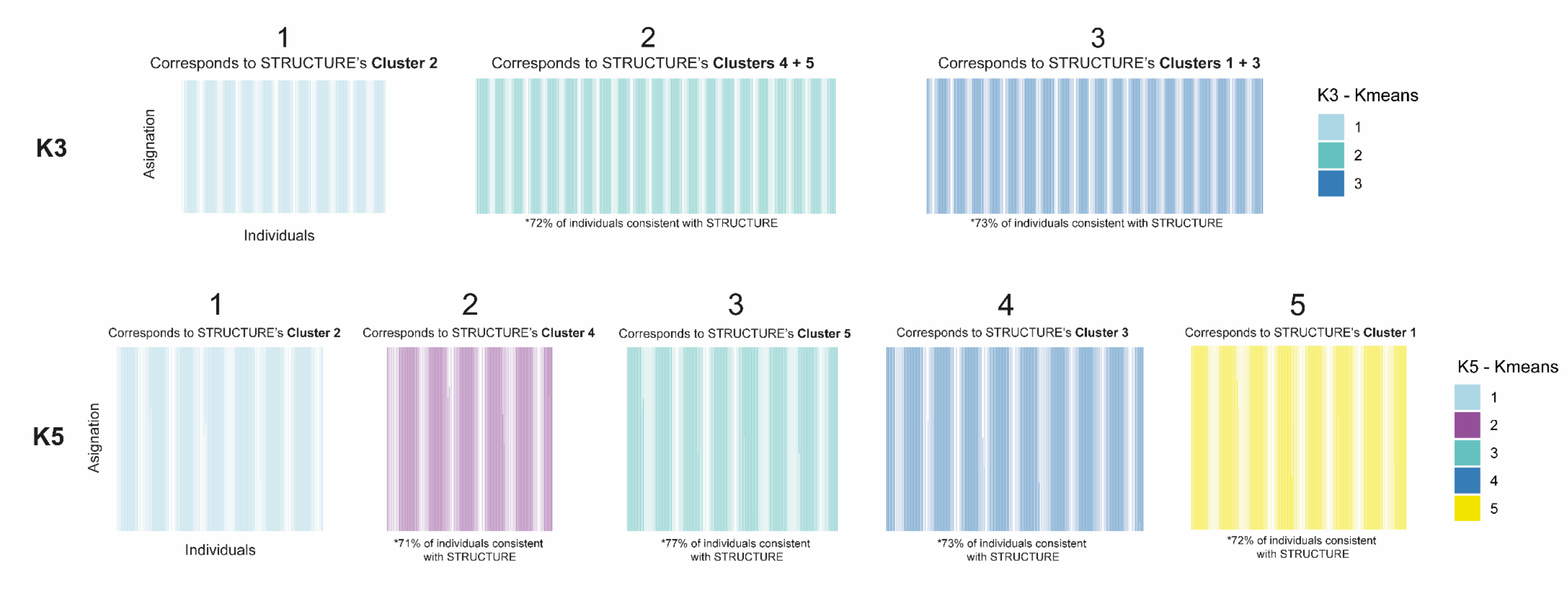

Fig. S2. K-means clustering analysis (*K* = 1–7). Plots show individual assignment probabilities for *K* = 3 (top) and *K* = 5 (bottom). Each vertical bar represents one individual, and colours indicate membership in the different clusters. Headers detail the correspondence between K-means groups and STRUCTURE software clusters, while the percentages at the bottom indicate the level of consistency of K-means relative to STRUCTURE.

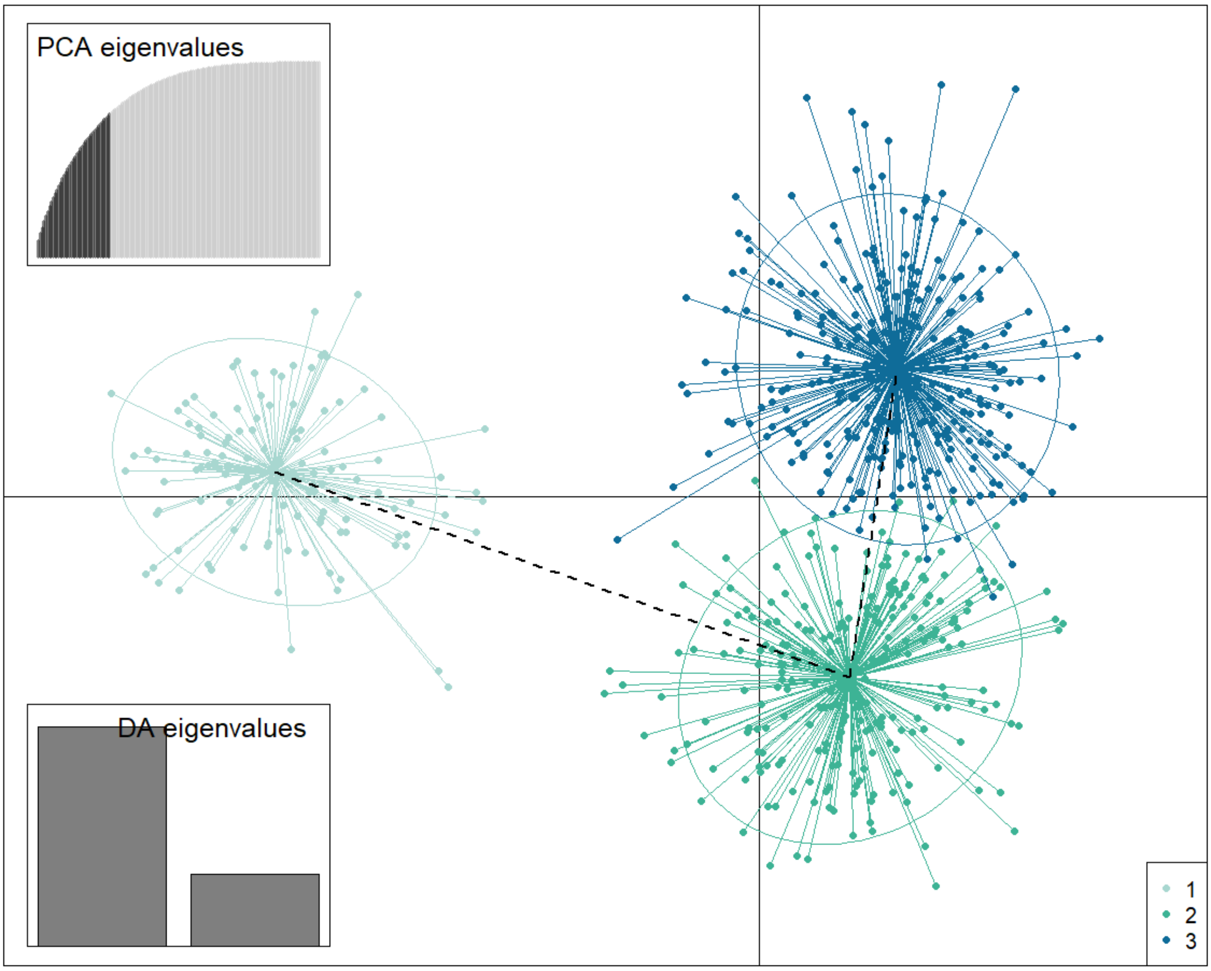

Fig. S3. Scatterplot of Discriminant Analysis of Principal Components (DAPC) at *K*=3. Dots represent individuals. No *a priori* assingnment of individuals to groups was applied. Clusters are defined by the following colours: (aquamarine) cluster 2-diploids; (emerald green) clusters 4 and 5 (the tetraploids plus the hexaploids extended to the north-eastern part of the region considered); and (dark blue) clusters 3 and 1 (the hexaploids represented in the southern and in the north-western part of the Balkan Peninsula).

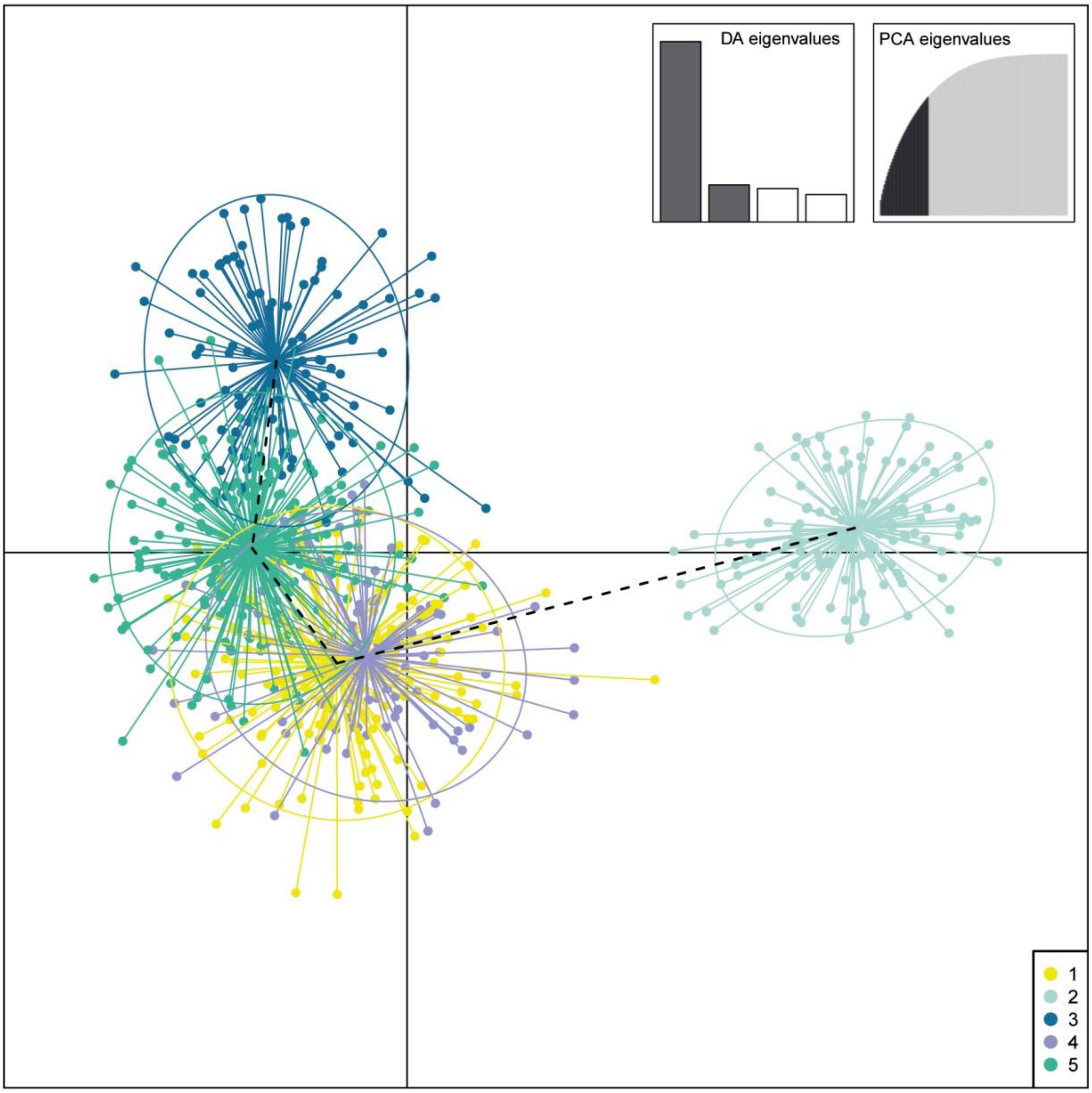

Fig. S4. Scatterplot of Discriminant Analysis of Principal Components (DAPC) at *K*=5. Dots represent individuals. No *a priori* assignment of individuals to groups was applied. Clusters are defined by the following colours: (yellow) cluster 1-southern genetic-geographic group, hexaploids; (aquamarine) cluster 2-diploids; (dark blue) cluster 3-western genetic-geographic group, hexaploids; (violet) cluster 4-central western tetraploids; and (emerald green) cluster 5-northern genetic-geographic group of hexaploids, plus three northeastern tetraploid populations.

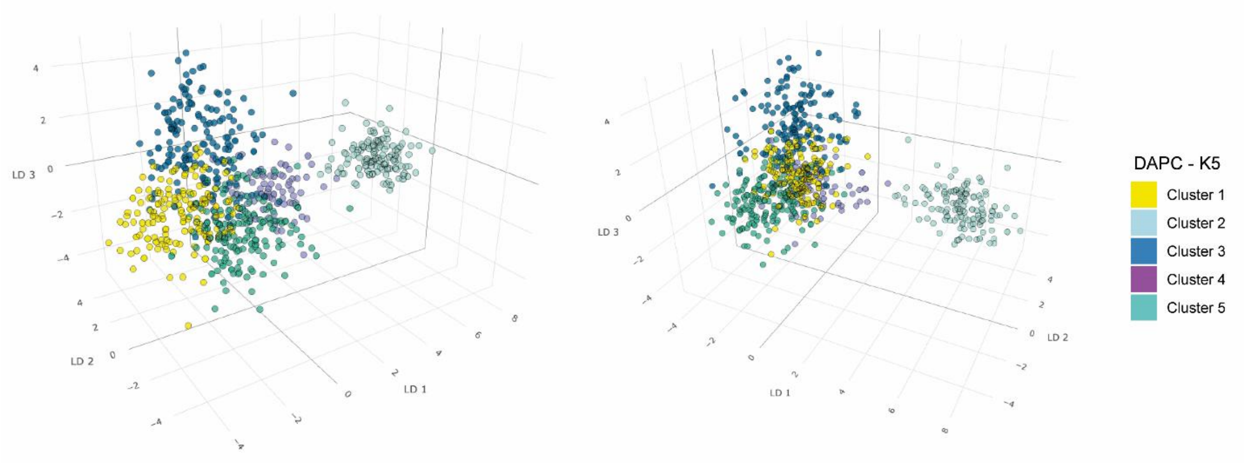

Fig. S5. 3D representation of the Discriminant Analysis of Principal Components (DAPC) for *K* = 5. The scatter plots illustrate the genetic clustering of individuals based on the first three linear discriminants (LD1, LD2, and LD3). Each point represents an individual, with colours indicating membership in one of the five identified clusters (Clusters 1–5). An interactive HTML version of this figure is available in the supplementary material as FIGS5_3D_DAPC.html.

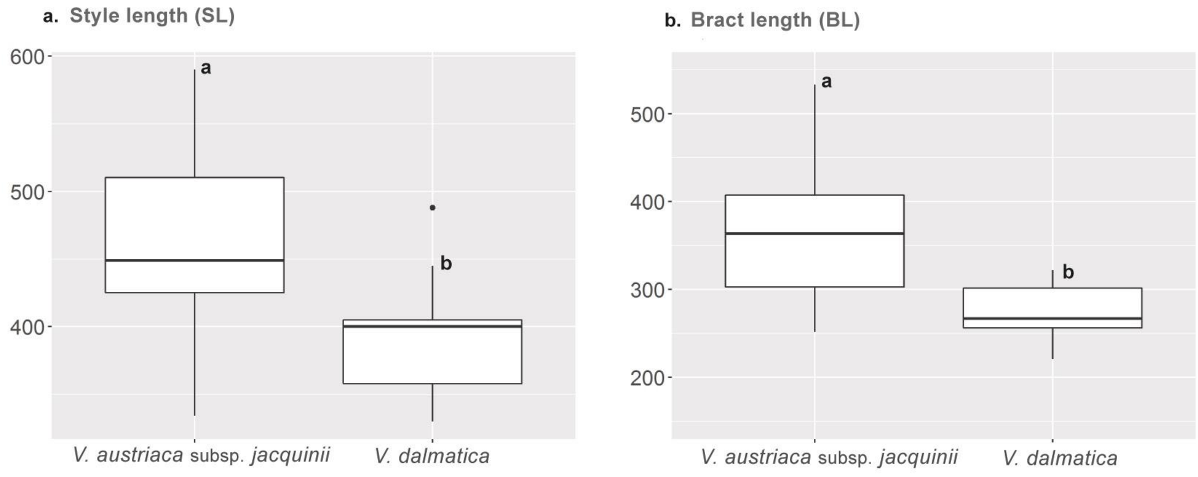

Fig. S6. Boxplots representing the variation and median values of the most discriminant characters to distinguish between *V. dalmatica* and *V. austriaca* ssp. *jacquinii*. **(a)** Style length (SL) and **(b)** Bract length (BL).

Table S1. Voucher information of the populations of *Veronica* included in this study. *Ploidy level obtained from López-González et al. (2021).

**Population**	**No. of indivs.**	**Ploidy level**	**Collection country, locality and altitude**	**Latitude, Longitude**	**Voucher[a] (Collector, date)**
Pop. 1	20	6*x*	Italy. Trieste. Aurisina surroundings, way to Trieste. 177 m.	45.74253N, 13.68331E	SALA155844 (Padilla-García et al., 17-VI-2014)
Pop. 2	20	6*x*	Italy. Trieste. Between Monrupino and Fernetti. 329 m.	45.71117N, 13.81217E	SALA155838 and SALA155880 (Martínez-Ortega et al., 18-VI-2014)
Pop. 3	15	6*x*	Croatia. Between Josipdol and Plitvička Jezera National Park, Lička Jesenica. 513 m.	45.00683N, 15.42800E	SALA149380 (Martínez-Ortega et al., 7-VII-2010)
Pop. 4	14	6*x*	Austria. Krems, between Weissenkirchen and Dürnstein. 259 m.	48.40502N, 15.51789E	SALA149043 (Rojas-Andrés et al., 22-VII-2011)
Pop. 5	18	6*x*	Croatia. Lika-Senj. Between Prijeboj and Ličko. 670 m.	44.84569N, 15.68406E	SALA149339 (Martínez-Ortega et al., 7-VII-2010)
Pop. 6	17	6*x*	Croatia. Zadar, Gračac, Crnopac. 761 m.	44.25061N, 15.80986E	SALA149372, SALA149373 and SALA149374 (Rojas-Andrés et al., 8-VII-2010)
Pop. 7	15	6*x*	Austria. Kalksburg. On the climb from the street "In der Klausen". 338 m.	48.14111N, 16.24728E	SALA149383 (Martínez-Ortega et al., 24-VII-2011)
Pop. 8	16	4*x*	Croatia. Split-Dalmatia, Split, Klis, Markezina Greda, towards Sv. Jure. 559 m.	43.56703N, 16.48278E	SALA149341 (Andrés-Sánchez et al., 9-VII-2010)
Pop. 9	16	4*x*	Bosnia and Herzegovina. West Herzegovina, Ljubuški. 169 m.	43.21289N, 17.53342E	SALA149353 (Rojas-Andrés et al., 11-VII-2010)
Pop. 10	15	6*x*	Bosnia-Herzegovina. Travnik, Vlašić. 1482 m.	44.27483N, 17.59997E	SALA149389 (Andrés-Sánchez et al., 12-VII-2010)

Population	No. of indivs.	Ploidy level	Collection country, locality and altitude	Latitude, Longitude	Voucher[a] (Collector, date)
Pop. 11	6	4*x*	Bosnia and Herzegovina. Herzegovina-Neretva, Potoci, Porim planina, Rujiste. 1112 m.	43.46342N, 17.95917E	SALA149041 (Rojas-Andrés et al., 11-VII-2010)
Pop. 12	17	2*x*	Croatia. Dubrovnik-Neretva, Dubrovnik, Gromača. 320 m.	42.72444N, 18.01778E	SALA149039 (Andrés-Sánchez et al., 14-VII-2010)
Pop. 13	15	2*x*	Croatia. Dubrovnik-Neretva, Dubrovnik, between Sumet and Gornji Brgat. 212 m.	42.64408N, 18.14644E	SALA149286 (Andrés-Sánchez et al., 15-VII-2010)
Pop. 14	15	2*x*	Bosnia and Herzegovina. Trebinje, between Trebinje and Dubrovnik. 282 m.	42.68392N, 18.29700E	SALA149284 (Andrés-Sánchez et al., 14-VII-2010)
Pop. 15	17	4*x*	Bosnia and Herzegovina. Municipality of Pale, Sarajevo, Trebević. 1146 m.	43.83508N, 18.43531E	SALA149355 (Andrés-Sánchez et al., 12-VII-2010)
Pop. 16	17	2*x*	Bosnia and Herzegovina. Trevinje, between Tjentište and Gacko. 1085 m.	43.18547N, 18.56603E	SALA149274 (Andrés-Sánchez et al., 13-VII-2010)
Pop. 17	15	4*x*	Bosnia and Herzegovina. Foča, Sutjeska, Tjentište. 670 m.	43.36886N, 18.71092E	SALA149354 (Martínez-Ortega et al., 13-VII-2010)
Pop. 18	20	2*x*	Montenegro. Kotor, Lovćen. 904 m.	42.41803N, 18.79411E	SALA149292 and SALA149296 (Andrés-Sánchez et al., 15-VII-2010)
Pop. 19	16	4*x*	Montenegro. Plužine, valley of the river Piva, on the way to Borkovici. 1039 m.	43.05572N, 18.89081E	SALA149352 (Rojas-Andrés et al., 17-VII-2010)
Pop. 20	20	6*x*	Montenegro. Mts. Treskavac, between Borkovici and Boricje. 1383 m.	43.10678N, 18.90789E	SALA149357 to SALA149362 and SALA149369 (Rojas-Andrés et al., 17-VII-2010)
Pop. 21	20	2*x*	Montenegro. Bar, between Sutorman and Karuci, Rumija Planina. 738 m.	42.16105N, 19.09708E	SALA157017 (Martínez-Ortega et al., 9-VI-2015)

Population	No. of indivs.	Ploidy level	Collection country, locality and altitude	Latitude, Longitude	Voucher[a] (Collector, date)
Pop. 22	15	6*x*	Montenegro. Ljubišnja Planina, Bobovo. 1475 m.	43.27097N, 19.14983E	SALA149390 (Andrés-Sánchez et al., 18-VII-2010)
Pop. 23	18	2*x*	Montenegro. Žabljak, Žabljak, near the village. 1392 m.	43.16378N, 19.15008E	SALA149293 and SALA149287 (Andrés-Sánchez et al., 182-VII-2010)
Pop. 24	4	2*x*	Albania. Lezhë, Lezhe, near Fishte. 56 m.	41.89112N, 19.67781E	SALA157035 (Martínez-Ortega et al., 17-VI-2015)
Pop. 25	20	2*x*	Montenegro. Andrijevica, Andrijevica towards Kolasin. 884 m.	42.74523N, 19.77552E	SALA157016 (Martínez-Ortega et al., 8-VI-2015)
Pop. 26	4	6*x*	Hungary. Heves. Gyöngyös, Gyöngyösi Sárhegy. 482 m.	47.81003N, 20.00075E	SALA155883 (Rojas-Andrés et al., 25-VII-2014)
Pop. 27	10	6*x*	Slovakia. Muráň. 649 m.	48.75112N, 20.04861E	SALA157057 (Martínez-Ortega et al., 16-VII-2014)
Pop. 28	20	6*x*	Slovakia. Letanovce. Path from Trsteny Potola up to mountain Ihrik. 619 m.	48.95525N, 20.43775E	SALA153002 (Rojas-Andrés et al., 17-VII-2014)
Pop. 29	10	6*x*	Serbia. Devojački Bunar, Vladimirovac. 155 m.	45.003N, 20.93919E	SALA149392 (Andrés-Sánchez et al., 27-VII-2010)
Pop. 30	20	6*x*	Republic of North Macedonia. Tetovo, between Stenče and Gurgurnica. 1291 m.	41.87981N, 21.04070E	SALA155841 (Rojas-Andrés et al., 6-VII-2014)
Pop. 31	20	6*x*	Republic of North Macedonia. Studeničani, Dračevo, between Crvena Voda and Paligrad. 663 m.	41.86184N, 21.45830E	SALA155840 (Rojas-Andrés et al., 12-VII-2014)
Pop. 32	18	6*x*	Serbia. Pomoravlje, between Krepoljin and Despotovac. 286 m.	44.12586N, 21.50622E	SALA149365 (Rojas-Andrés et al., 26-VII-2010)

Population	No. of indivs.	Ploidy level	Collection country, locality and altitude	Latitude, Longitude	Voucher[a] (Collector, date)
Pop. 33	16	6*x*	Republic of North Macedonia. Prilep, Sivec, near the marble quarry. 815 m.	41.40714N, 21.60386E	SALA155839 (López-González et al., 10-VII-2014)
Pop. 34	20	6*x*	Slovakia. Košice, distr. Trebišov, surroundings of Černochov. 245 m.	48.43022N, 21.70586E	SALA157058 (López-González et al., 18-VII-2014)
Pop. 35	8	6*x*	Serbia. Zaječar. Sokobanja, Rtanj. Base of mount Rtanj. 498 m.	43.78564N, 21.91822E	SALA149364 (Rojas-Andrés et al., 22-VII-2010)
Pop. 36	15	6*x*	Republic of North Macedonia. Kavadarci, Kozjak Planina, Majden. 751 m.	41.159N, 21.944E	SALA149391 (Andrés-Sánchez et al., 24-VII-2010)
Pop. 37	20	6*x*	Slovakia. Košice, Vinné, way up to castle Viniansky Hrad. 164 m.	48.81709N, 21.94442E	SALA155836 (López-González et al., 18-VII-2014)
Pop. 38	10	6*x*	Greece. Imathia, Mt. Vermion. 1459 m.	40.55183N, 22.02356E	SALA149387 (Santos-Vicente et al., 25-VI-2009)
Pop. 39	20	4*x*	Romania. Baile Herculane, near the town next to 67D road. 223 m.	44.87198N, 22.41441E	SALA155842 (López-González et al., 22-VII-2014)
Pop. 40	10	6*x*	Bulgaria. Pernik, close to Tran, between Vukan and Konsturintsi. 786 m.	42.77236N, 22.59106E	SALA149375 (Martínez-Ortega et al., 14-VI-2009)
Pop. 41	10	6*x*	Bulgaria. Sofia, path up to Vitosha. 1578 m.	42.58992N, 23.31244E	SALA149366 (Rojas-Andrés et al., 15-VI-2009)
Pop. 42	10	6*x*	Greece. Chalkidiki, mount Cholomontas (Ypsizon). 503 m.	40.49519N, 23.45344E	SALA149379 (Martínez-Ortega et al., 24-VI-2009)
Pop. 43	20	6*x*	Romania. Cluj Napoca. Fanatele Clujului. 503 m.	46.83769N, 23.62064E	SALA155862 (Martínez-Ortega et al., 20-VII-2014)

Population	No. of indivs.	Ploidy level	Collection country, locality and altitude	Latitude, Longitude	Voucher[a] (Collector, date)
Pop. 44	9	6*x*	Romania. Alba. Mănărade. 503 m.	46.14553N, 23.97814E	SALA155862 (Martínez-Ortega et al., 20-VII-2014)
Pop. 45	17	4*x*	Romania. Alba. Valea Lungă, towards Tauri monastery. 316 m.	46.140583N, 24.057278E	SALA157051 (Martínez-Ortega et al., 20-VII-2014)
Pop. 46	12	6*x*	Bulgaria. Pazardzhik, between Batak and Beglika. 1549 m.	41.84086N, 24.14664E	SALA149367and SALA149368 (Rojas-Andrés et al., 17-VI-2009)
Pop. 47	20	4*x*	Romania. Sibiu, Sura Mare. Surroundings of Hamba. 494 m.	45.87133N, 24.20411E	SALA155843 (Rojas-Andrés et al., 21-VII-2014)
Pop. 48	10	6*x*	Bulgaria. Plovdiv, between Oreshets and Dobrostan. 1109 m.	41.89561N, 24.93278E	SALA149384 (Santos-Vicente et al., 18-VI-2009)
Pop. 49	10	6*x*	Greece. Northwards Komotini, between Pandrossos and Symvola. 302 m.	41.18542N, 25.42639E	SALA149378 (Martínez-Ortega et al., 24-VI-2009)
Pop. 50	11	6*x*	Bulgaria. Sliven. 689 m.	42.72258N, 26.29439E	SALA149370 (Rojas-Andrés et al., 20-VI-2009)

[a] Herbarium acronym and herbarium code

Table S2. Leaf and fruit characters measured, and abbreviations used along the text.

**Leaf character abbreviation***	**Morphological leaf character**	
**LI**	Length of trichomes (measured at leaf margin)	
**DI**	Density of indumentum (measured at leaf margin)	
**LM**	Length of trichomes (measured 0.5 cm inwards from leaf margin)	
**DM**	Density of indumentum (measured 0.5 cm inwards from leaf margin)	
**MLW**	Width	Maximum width
**WMP**		Middle part
**TLW**		Entire terminal part
**FTW**		First tooth
**WF1**		First division/segment (bipinnatisect leaf)
**STW**		Second tooth
**WF2**		First tooth of the second segment (bipinnatisect leaf)
**LL**	Length	Total
**FTL**		First tooth/segment
**LF1**		First division/segment (bipinnatisect leaf)
**STL**		Second tooth/segment
**LF2**		First tooth of the second segment (bipinnatisect leaf)
**PL**		Petiole
**DBMW**	Distance between the leaf base and the maximum width line	
**DLAU**	Distance between the leaf apex and the uppermost teeth	
**NT**	Number of teeth per hemilimb	
**Fruit character abbreviation**	**Morphological fruit character**	
**SL**	Style length	
**CL**	Maximum capsule length	
**CW**	Maximum capsule width	
**PL**	Pedicel length	
**BL**	Bract length	

* The measurements were taken from a leaf at the medium part of the stem (medium leaves) and from a leaf from the apical shoot. Along the text, the abbreviations corresponding to each morphological leaf character measured will add an “M” (for the medium leaves) or an “S” (for the leaves of the apical shoot) at their respective word endings.

Table S3. Newly generated genome size estimations and ploidy level inferences (1Cx, 1C and 2C-values), internal standards, sample and standard CV for the individuals included in the present study. *Ploidy level obtained from López-González et al. (2021).

**Population**	**Individual/s**	**no. of inds.**	**Ploidy**	**1Cx**	**1C**	**2C**	**Standard**	**CV sample**	**CV standard**
Pop. 1	1->5	5	**6**	0.59	1.78	3.55	*Raphanus sativus*	4.80	3.59
Pop. 1	6->10	5	**6**	0.58	1.74	3.48	*Raphanus sativus*	4.08	3.2
Pop. 1	11->15	5	**6**	0.58	1.73	3.46	*Raphanus sativus*	3.62	3.89
Pop. 1	16->20	5	**6**	0.58	1.73	3.47	*Raphanus sativus*	4.21	3.3
Pop. 2	1,4,5	3	**6**	0.59	1.77	3.53	*Raphanus sativus*	3.22	3.66
Pop. 2	2	1	**6**	0.58	1.74	3.48	*Raphanus sativus*	2.51	3.32
Pop. 2	3	1	**6**	0.59	1.76	3.51	*Raphanus sativus*	3.35	2.43
Pop. 2	6->9	4	**6**	0.58	1.75	3.50	*Raphanus sativus*	2.59	2.83
Pop. 2	10->14	5	**6**	0.58	1.75	3.49	*Raphanus sativus*	2.95	3.38
Pop. 2	15->19	5	**6**	0.59	1.76	3.53	*Raphanus sativus*	3.06	2.65
Pop. 2	20,22,23	3	**6**	0.58	1.73	3.46	*Raphanus sativus*	3.88	3.77
Pop. 2	21	1	**6**	0.60	1.79	3.59	*Raphanus sativus*	3.21	3.25
Pop. 3*			**6**						
Pop. 4	1	1	**6**	0.66	1.98	3.97	*Solanum lycopersicum*	3.22	2.72
Pop. 4	2	1	**6**	0.64	1.92	3.85	*Solanum lycopersicum*	4.82	3.46
Pop. 4	3	1	**6**	0.64	1.91	3.81	*Solanum lycopersicum*	3.28	4.33
Pop. 4	4	1	**6**	0.66	1.99	3.98	*Solanum lycopersicum*	4.75	2.81
Pop. 4	5	1	**6**	0.66	1.98	3.96	*Solanum lycopersicum*	4.50	2.86
Pop. 4	6	1	**6**	0.64	1.93	3.87	*Solanum lycopersicum*	3.48	3.39
Pop. 4	7	1	**6**	0.64	1.93	3.86	*Solanum lycopersicum*	3.93	3.12
Pop. 4	8	1	**6**	0.65	1.94	3.88	*Solanum lycopersicum*	3.40	2.65
Pop. 4	9	1	**6**	0.65	1.94	3.88	*Solanum lycopersicum*	5.77	3.81

**Population**	**Individual/s**	**no. of inds.**	**Ploidy**	**1Cx**	**1C**	**2C**	**Standard**	**CV sample**	**CV standard**
Pop. 4	10	1	**6**	0.64	1.93	3.87	*Solanum lycopersicum*	4.48	2.64
Pop. 4	11	1	**6**	0.65	1.94	3.88	*Solanum lycopersicum*	4.24	3.9
Pop. 4	12	1	**6**	0.65	1.94	3.88	*Solanum lycopersicum*	4.53	2.74
Pop. 4	13	1	**6**	0.66	1.98	3.96	*Solanum lycopersicum*	4.34	3.97
Pop. 4	14	1	**6**	0.65	1.94	3.88	*Solanum lycopersicum*	3.72	5.02
Pop. 5*			**6**						
Pop. 6*			**6**						
Pop. 7	1	1	**6**	0.67	2.01	4.03	*Solanum lycopersicum*	3.09	2.97
Pop. 7	2	1	**6**	0.66	1.98	3.97	*Solanum lycopersicum*	3.48	3.09
Pop. 7	3	1	**6**	0.64	1.92	3.83	*Solanum lycopersicum*	3.38	3.39
Pop. 7	4	1	**6**	0.64	1.93	3.86	*Solanum lycopersicum*	3.39	3.74
Pop. 7	5	1	**6**	0.65	1.94	3.89	*Solanum lycopersicum*	3.31	3
Pop. 7	6	1	**6**	0.64	1.93	3.86	*Solanum lycopersicum*	3.37	3.45
Pop. 7	7	1	**6**	0.63	1.88	3.76	*Solanum lycopersicum*	4.65	4.65
Pop. 7	8	1	**6**	0.66	1.99	3.98	*Solanum lycopersicum*	3.56	2.89
Pop. 7	9	1	**6**	0.64	1.92	3.84	*Solanum lycopersicum*	4.38	3.9
Pop. 7	10	1	**6**	0.66	1.99	3.98	*Solanum lycopersicum*	3.42	3.15
Pop. 7	11	1	**6**	0.65	1.94	3.88	*Solanum lycopersicum*	3.78	4.49
Pop. 7	12	1	**6**	0.67	2.00	3.99	*Solanum lycopersicum*	3.79	2.84
Pop. 7	13	1	**6**	0.66	1.98	3.96	*Solanum lycopersicum*	3.25	2.68
Pop. 7	14	1	**6**	0.67	2.02	4.05	*Solanum lycopersicum*	3.70	2.8
Pop. 7	15	1	**6**	0.67	2.00	4.00	*Solanum lycopersicum*	3.25	2.92
Pop. 8*			**4**						
Pop. 9*			**4**						
Pop. 10*			**6**						

**Population**	**Individual/s**	**no. of inds.**	**Ploidy**	**1Cx**	**1C**	**2C**	**Standard**	**CV sample**	**CV standard**
Pop. 11*			**4**						
Pop. 12*			**2**						
Pop. 13*			**2**						
Pop. 14*			**2**						
Pop. 15*			**4**						
Pop. 16*			**2**						
Pop. 17*			**4**						
Pop. 18*			**2**						
Pop. 19*			**4**						
Pop. 20	1	1	**6**	0.66	1.98	3.97	*Pisum sativum* cv. Ctirad	7.09	5.09
Pop. 20	2	1	**6**	0.64	1.92	3.85	*Pisum sativum* cv. Ctirad	7.01	5.09
Pop. 20	3	1	**6**	0.66	1.99	3.98	*Pisum sativum* cv. Ctirad	7.52	5.84
Pop. 20	4	1	**6**	0.66	1.97	3.94	*Pisum sativum* cv. Ctirad	4.73	1.83
Pop. 20	5	1	**6**	0.66	1.99	3.98	*Pisum sativum* cv. Ctirad	4.91	1.75
Pop. 20	6	1	**6**	0.65	1.94	3.89	*Pisum sativum* cv. Ctirad	3.89	1.54
Pop. 20	7	1	**6**	0.63	1.89	3.79	*Zea mays* cv. CE-777	5.10	2.13
Pop. 20	10	1	**6**	0.64	1.91	3.82	*Zea mays* cv. CE-777	4.73	2.49
Pop. 20	13	1	**6**	0.59	1.78	3.55	*Pisum sativum* cv. Kleine Rheinländerin	4.17	1.53
Pop. 20	8,9,11,12	4	**6**	0.65	1.94	3.87	*Solanum lycopersicum*	3.50	3.1
Pop. 20	14->17	4	**6**	0.71	2.12	4.24	*Solanum lycopersicum*	3.48	2.95
Pop. 20	18->20	3	**6**	0.67	2.02	4.03	*Solanum lycopersicum*	4.79	2.99
Pop. 21*			**2**						
Pop. 22	1->4	4	**6**	0.70	2.09	4.18	*Solanum lycopersicum*	4.23	2.86
Pop. 22	5,8,9,12	4	**6**	0.69	2.07	4.13	*Solanum lycopersicum*	3.69	2.24
Pop. 22	6	1	**6**	0.63	1.90	3.79	*Zea mays* cv. CE-777	2.46	1.20

**Population**	**Individual/s**	**no. of inds.**	**Ploidy**	**1Cx**	**1C**	**2C**	**Standard**	**CV sample**	**CV standard**
Pop. 22	7	1	**6**	0.61	1.83	3.66	*Solanum pseudocapsicum*	5.24	4.52
Pop. 22	10	1	**6**	0.61	1.83	3.67	*Pisum sativum* cv. Kleine Rheinländerin	3.82	2.13
Pop. 22	11	1	**6**	0.63	1.89	3.79	*Zea mays* cv. CE-777	2.35	1.36
Pop. 22	13->15	3	**6**	0.68	2.05	4.11	*Solanum lycopersicum*	3.77	2.73
Pop. 23*			**2**						
Pop. 24*			**2**						
Pop. 25*			**2**						
Pop. 26	1	1	**6**	0.65	1.95	3.89	*Raphanus sativus*	2.32	2.4
Pop. 26	2	1	**6**	0.65	1.95	3.90	*Raphanus sativus*	3.03	2.33
Pop. 26	3	1	**6**	0.63	1.88	3.76	*Raphanus sativus*	3.48	2.76
Pop. 26	4	1	**6**	0.65	1.94	3.87	*Raphanus sativus*	3.05	2.44
Pop. 27	1	1	**6**	0.62	1.85	3.70	*Raphanus sativus*	2.39	2.79
Pop. 27	2	1	**6**	0.62	1.86	3.71	*Raphanus sativus*	2.43	2.79
Pop. 27	3->6	4	**6**	0.60	1.80	3.60	*Raphanus sativus*	2.83	2.76
Pop. 27	7	1	**6**	0.61	1.84	3.67	*Raphanus sativus*	2.19	2.65
Pop. 27	8,9	2	**6**	0.62	1.85	3.70	*Raphanus sativus*	3.03	2.65
Pop. 27	10	1	**6**	0.60	1.80	3.60	*Raphanus sativus*	1.86	2.52
Pop. 28	1	1	**6**	0.63	1.88	3.76	*Raphanus sativus*	2.30	2.69
Pop. 28	2->4	3	**6**	0.60	1.79	3.58	*Raphanus sativus*	3.04	3.49
Pop. 28	5	1	**6**	0.62	1.87	3.73	*Raphanus sativus*	2.04	2.61
Pop. 28	6,7,8,10	4	**6**	0.61	1.82	3.65	*Raphanus sativus*	5.65	3.62
Pop. 28	9	1	**6**	0.64	1.92	3.83	*Raphanus sativus*	3.14	3.46
Pop. 28	11->15	5	**6**	0.60	1.80	3.60	*Raphanus sativus*	2.93	4.18
Pop. 28	16->20	5	**6**	0.62	1.85	3.71	*Raphanus sativus*	3.72	3.02
Pop. 29	1,3,4,6	4	**6**	0.66	1.99	3.98	*Solanum lycopersicum*	3.88	3

**Population**	**Individual/s**	**no. of inds.**	**Ploidy**	**1Cx**	**1C**	**2C**	**Standard**	**CV sample**	**CV standard**
Pop. 29	2	1	**6**	0.63	1.89	3.78	*Zea mays* cv. CE-777	4.16	2.94
Pop. 29	5	1	**6**	0.62	1.86	3.71	*Zea mays* cv. CE-777	5.13	2.99
Pop. 29	7	1	**6**	0.62	1.86	3.73	*Zea mays* cv. CE-777	3.74	2.42
Pop. 29	8->10	3	**6**	0.63	1.89	3.79	*Solanum lycopersicum*	4.76	3
Pop. 30	1->5	5	**6**	0.61	1.83	3.66	*Raphanus sativus*	4.74	4.13
Pop. 30	6->10	5	**6**	0.62	1.87	3.74	*Raphanus sativus*	4.07	3.33
Pop. 30	11->15	5	**6**	0.62	1.87	3.74	*Raphanus sativus*	4.34	3.63
Pop. 30	16->20	5	**6**	0.62	1.85	3.71	*Raphanus sativus*	3.80	4.18
Pop. 31	1->5	5	**6**	0.58	1.75	3.50	*Raphanus sativus*	3.99	3.7
Pop. 31	6->10	5	**6**	0.57	1.70	3.40	*Raphanus sativus*	3.43	3.29
Pop. 31	11->15	5	**6**	0.57	1.70	3.40	*Raphanus sativus*	3.27	3.2
Pop. 31	16->20	5	**6**	0.57	1.70	3.40	*Raphanus sativus*	3.18	3.32
Pop. 32	1	1	**6**	0.61	1.83	3.65	*Solanum lycopersicum*	4.18	4.5
Pop. 32	2	1	**6**	0.63	1.90	3.81	*Solanum lycopersicum*	4.79	4.6
Pop. 32	3	1	**6**	0.61	1.84	3.68	*Solanum lycopersicum*	4.99	3.97
Pop. 32	4	1	**6**	0.62	1.86	3.72	*Solanum lycopersicum*	4.89	4.65
Pop. 32	5	1	**6**	0.62	1.86	3.72	*Solanum lycopersicum*	4.42	4.26
Pop. 32	6	1	**6**	0.65	1.96	3.91	*Solanum lycopersicum*	4.74	4.62
Pop. 32	7	1	**6**	0.64	1.93	3.86	*Solanum lycopersicum*	4.26	4.19
Pop. 32	8	1	**6**	0.64	1.93	3.87	*Solanum lycopersicum*	4.16	4.69
Pop. 32	9	1	**6**	0.64	1.92	3.83	*Solanum lycopersicum*	4.94	4.22
Pop. 32	10	1	**6**	0.66	1.97	3.93	*Solanum lycopersicum*	4.20	3.84
Pop. 32	11	1	**6**	0.65	1.96	3.92	*Solanum lycopersicum*	3.44	4.23
Pop. 32	12	1	**6**	0.56	1.67	3.33	*Solanum lycopersicum*	3.27	5.04
Pop. 32	13	1	**6**	0.61	1.84	3.69	*Solanum lycopersicum*	4.36	4.64

**Population**	**Individual/s**	**no. of inds.**	**Ploidy**	**1Cx**	**1C**	**2C**	**Standard**	**CV sample**	**CV standard**
Pop. 32	14	1	**6**	0.62	1.87	3.74	*Solanum lycopersicum*	4.86	3.65
Pop. 32	15	1	**6**	0.63	1.90	3.80	*Solanum lycopersicum*	4.78	3.39
Pop. 32	16	1	**6**	0.61	1.84	3.69	*Solanum lycopersicum*	4.43	4.62
Pop. 32	17	1	**6**	0.61	1.82	3.65	*Solanum lycopersicum*	4.50	4.56
Pop. 32	18	1	**6**	0.65	1.94	3.89	*Solanum lycopersicum*	4.69	3.8
Pop. 33	1,2,5,6	4	**6**	0.59	1.77	3.55	*Raphanus sativus*	2.94	3.34
Pop. 33	3	1	**6**	0.60	1.79	3.58	*Raphanus sativus*	2.55	2.51
Pop. 33	4	1	**6**	0.61	1.82	3.64	*Raphanus sativus*	2.20	2.73
Pop. 33	7->10	4	**6**	0.61	1.82	3.64	*Raphanus sativus*	3.12	3.1
Pop. 33	11	1	**6**	0.60	1.81	3.61	*Raphanus sativus*	1.86	3.03
Pop. 33	12->16	5	**6**	0.58	1.74	3.49	*Raphanus sativus*	3.31	2.55
Pop. 34	1->4	4	**6**	0.62	1.86	3.73	*Raphanus sativus*	3.15	3.27
Pop. 34	5,6,8,11,12	5	**6**	0.61	1.84	3.69	*Raphanus sativus*	2.92	3.91
Pop. 34	7	1	**6**	0.64	1.91	3.82	*Raphanus sativus*	2.55	3.28
Pop. 34	9	1	**6**	0.62	1.86	3.72	*Raphanus sativus*	3.25	3.53
Pop. 34	10	1	**6**	0.62	1.85	3.70	*Raphanus sativus*	2.39	3.56
Pop. 34	13->17	4	**6**	0.60	1.80	3.60	*Raphanus sativus*	2.34	2.98
Pop. 34	18->20	3	**6**	0.60	1.80	3.60	*Raphanus sativus*	3.47	3.4
Pop. 35	1,3,4	3	**6**	0.67	2.01	4.02	*Solanum lycopersicum*	4.92	3.28
Pop. 35	2	1	**6**	0.61	1.84	3.69	*Zea mays* cv. CE-777	6.36	4.31
Pop. 35	5,7,8	3	**6**	0.62	1.85	3.70	*Solanum lycopersicum*	4.34	3.21
Pop. 35	6	1	**6**	0.62	1.87	3.74	*Zea mays* cv. CE-777	3.18	2.06
Pop. 36	1,3,5	3	**6**	0.68	2.04	4.09	*Solanum lycopersicum*	3.75	3.61
Pop. 36	2	1	**6**	0.65	1.94	3.89	*Zea mays* cv. CE-777	4.75	2.03
Pop. 36	4	1	**6**	0.64	1.91	3.81	*Zea mays* cv. CE-777	3.55	1.86

**Population**	**Individual/s**	**no. of inds.**	**Ploidy**	**1Cx**	**1C**	**2C**	**Standard**	**CV sample**	**CV standard**
Pop. 36	6	1	**6**	0.71	2.13	4.25	*Raphanus sativus*	4.85	3.71
Pop. 36	7	1	**6**	0.68	2.03	4.05	*Raphanus sativus*	3.92	3.95
Pop. 36	8	1	**6**	0.61	1.82	3.64	*Raphanus sativus*	3.76	3.52
Pop. 36	9	1	**6**	0.63	1.89	3.77	*Solanum pseudocapsicum*	4.94	1.65
Pop. 36	10	1	**6**	0.68	2.03	4.07	*Raphanus sativus*	5.21	4.14
Pop. 36	11	1	**6**	0.60	1.79	3.59	*Pisum sativum* cv. Kleine Rheinländerin	3.05	1.31
Pop. 36	12->15	4	**6**	0.65	1.94	3.88	*Solanum lycopersicum*	3.52	4.1
Pop. 37	1->5	5	**6**	0.61	1.84	3.68	*Raphanus sativus*	2.98	4.7
Pop. 37	6->10	5	**6**	0.60	1.80	3.59	*Raphanus sativus*	2.94	3.12
Pop. 37	11->15	5	**6**	0.61	1.84	3.69	*Raphanus sativus*	4.16	3.45
Pop. 37	16	1	**6**	0.60	1.80	3.60	*Raphanus sativus*	2.42	2.68
Pop. 37	17	1	**6**	0.61	1.84	3.67	*Raphanus sativus*	4.15	3.41
Pop. 37	18	1	**6**	0.60	1.80	3.60	*Raphanus sativus*	3.20	3.56
Pop. 37	19	1	**6**	0.64	1.93	3.86	*Raphanus sativus*	2.35	3.65
Pop. 37	20	1	**6**	0.61	1.82	3.64	*Raphanus sativus*	3.56	3.78
Pop. 38	1->4	4	**6**	0.62	1.86	3.73	*Solanum lycopersicum*	4.88	2.67
Pop. 38	5,8,10	3	**6**	0.69	2.08	4.17	*Solanum lycopersicum*	6.43	3.27
Pop. 38	6	1	**6**	0.63	1.88	3.77	*Zea mays* cv. CE-777	4.06	1.83
Pop. 38	7	1	**6**	0.62	1.86	3.71	*Zea mays* cv. CE-777	4.98	2.42
Pop. 38	9	1	**6**	0.64	1.92	3.84	*Zea mays* cv. CE-777	4.36	2.38
Pop. 39	1->4	4	**4**	0.56	1.12	2.23	*Raphanus sativus*	4.47	2.61
Pop. 39	5	1	**4**	0.57	1.14	2.28	*Raphanus sativus*	3.73	4.28
Pop. 39	6	1	**4**	0.59	1.17	2.34	*Raphanus sativus*	4.47	2.64
Pop. 39	7	1	**4**	0.58	1.15	2.30	*Raphanus sativus*	4.12	3.34
Pop. 39	8	1	**4**	0.58	1.16	2.32	*Raphanus sativus*	4.23	2.79

Population	Individual/s	no. of inds.	Ploidy	1Cx	1C	2C	Standard	CV sample	CV standard
Pop. 39	9	1	**4**	0.57	1.14	2.28	*Raphanus sativus*	3.55	2.55
Pop. 39	10->13	4	**4**	0.56	1.13	2.25	*Raphanus sativus*	3.80	2.74
Pop. 39	14->18	5	**4**	0.57	1.14	2.27	*Raphanus sativus*	4.50	3.29
Pop. 39	19->20	2	**4**	0.56	1.13	2.25	*Raphanus sativus*	4.71	3.22
Pop. 40	1	1	**6**	0.65	1.96	3.92	*Solanum lycopersicum*	3.27	3.34
Pop. 40	2	1	**6**	0.64	1.92	3.85	*Zea mays* cv. CE-777	3.07	1.41
Pop. 40	3	1	**6**	0.65	1.96	3.92	*Solanum lycopersicum*	3.66	3.96
Pop. 40	4	1	**6**	0.66	1.97	3.94	*Solanum lycopersicum*	2.68	2.41
Pop. 40	5	1	**6**	0.62	1.86	3.72	*Pisum sativum* cv. Kleine Rheinländerin	3.46	2.3
Pop. 40	6	1	**6**	0.66	1.99	3.99	*Solanum lycopersicum*	4.38	4.41
Pop. 40	7	1	**6**	0.62	1.86	3.72	*Solanum pseudocapsicum*	5.68	2.08
Pop. 40	8	1	**6**	0.65	1.95	3.89	*Solanum lycopersicum*	4.43	4.99
Pop. 40	9	1	**6**	0.58	1.73	3.46	*Solanum lycopersicum*	4.50	4.24
Pop. 40	10	1	**6**	0.65	1.96	3.92	*Solanum lycopersicum*	4.71	4.28
Pop. 41	1	1	**6**	0.66	1.99	3.99	*Zea mays* cv. CE-777	2.39	1.37
Pop. 41	2	1	**6**	0.68	2.03	4.05	*Solanum lycopersicum*	3.66	4.73
Pop. 41	3	1	**6**	0.68	2.05	4.10	*Solanum lycopersicum*	3.91	4.46
Pop. 41	4	1	**6**	0.70	2.10	4.20	*Solanum lycopersicum*	3.58	3.38
Pop. 41	5	1	**6**	0.68	2.05	4.10	*Solanum lycopersicum*	4.62	4.88
Pop. 41	6	1	**6**	0.70	2.10	4.19	*Solanum lycopersicum*	3.89	4.21
Pop. 41	7	1	**6**	0.68	2.03	4.07	*Solanum lycopersicum*	4.40	4.25
Pop. 41	8	1	**6**	0.67	2.00	3.99	*Zea mays* cv. CE-777	2.55	1.42
Pop. 41	9	1	**6**	0.62	1.87	3.74	*Solanum lycopersicum*	3.95	4.03
Pop. 41	10	1	**6**	0.64	1.92	3.84	*Pisum sativum* cv. Kleine Rheinländerin	2.94	1.28
Pop. 42	1	1	**6**	0.68	2.03	4.06	*Solanum lycopersicum*	4.14	3.67

**Population**	**Individual/s**	**no. of inds.**	**Ploidy**	**1Cx**	**1C**	**2C**	**Standard**	**CV sample**	**CV standard**
Pop. 42	2	1	**6**	0.67	2.02	4.04	*Solanum lycopersicum*	3.99	4.02
Pop. 42	3	1	**6**	0.61	1.83	3.65	*Solanum pseudocapsicum*	6.78	3.91
Pop. 42	4	1	**6**	0.63	1.89	3.78	*Zea mays* cv. CE-777	7.91	2.87
Pop. 42	5	1	**6**	0.68	2.04	4.08	*Solanum lycopersicum*	4.95	3.94
Pop. 42	6	1	**6**	0.67	2.02	4.04	*Solanum lycopersicum*	4.68	4.51
Pop. 42	7	1	**6**	0.68	2.03	4.05	*Solanum lycopersicum*	4.56	4.05
Pop. 42	8	1	**6**	0.68	2.03	4.05	*Solanum lycopersicum*	4.62	4.15
Pop. 42	9	1	**6**	0.62	1.86	3.73	*Solanum lycopersicum*	4.72	4.45
Pop. 42	10	1	**6**	0.69	2.07	4.14	*Solanum lycopersicum*	4.36	3.97
Pop. 43	1->5	5	**6**	0.62	1.85	3.70	*Raphanus sativus*	3.55	3.35
Pop. 43	6->10	5	**6**	0.62	1.87	3.73	*Raphanus sativus*	3.47	3.19
Pop. 43	11->15	5	**6**	0.59	1.78	3.57	*Raphanus sativus*	3.84	3.67
Pop. 43	16->20	5	**6**	0.60	1.80	3.60	*Raphanus sativus*	3.57	3.88
Pop. 44	1->5	5	**6**	0.60	1.81	3.63	*Raphanus sativus*	3.32	3.02
Pop. 44	6->9	4	**6**	0.62	1.87	3.74	*Raphanus sativus*	3.64	3.6
Pop. 45	1->6	6	**4**	0.66	1.32	2.64	*Raphanus sativus*	4.77	4.38
Pop. 45	13->17	5	**4**	0.62	1.23	2.46	*Raphanus sativus*	3.80	4.12
Pop. 45	7->12	6	**4**	0.62	1.25	2.49	*Raphanus sativus*	3.53	3.96
Pop. 46	1	1	**6**	0.65	1.96	3.92	*Solanum lycopersicum*	3.61	4.07
Pop. 46	2	1	**6**	0.63	1.90	3.80	*Solanum lycopersicum*	4.37	4.27
Pop. 46	3	1	**6**	0.64	1.92	3.84	*Solanum lycopersicum*	3.84	4.95
Pop. 46	4	1	**6**	0.62	1.87	3.73	*Solanum lycopersicum*	3.52	2.97
Pop. 46	5	1	**6**	0.65	1.95	3.90	*Solanum lycopersicum*	3.60	4.33
Pop. 46	6	1	**6**	0.65	1.95	3.90	*Solanum lycopersicum*	3.62	3.81
Pop. 46	7	1	**6**	0.64	1.92	3.83	*Solanum lycopersicum*	3.83	3.58

Population	Individual/s	no. of inds.	Ploidy	1Cx	1C	2C	Standard	CV sample	CV standard
Pop. 46	8	1	**6**	0.65	1.96	3.91	*Solanum lycopersicum*	3.69	4.53
Pop. 46	9	1	**6**	0.67	2.00	4.00	*Solanum lycopersicum*	4.03	3.97
Pop. 46	10	1	**6**	0.65	1.94	3.88	*Solanum lycopersicum*	5.32	3.78
Pop. 46	11	1	**6**	0.65	1.95	3.89	*Solanum lycopersicum*	3.09	4.54
Pop. 46	12	1	**6**	0.62	1.87	3.73	*Pisum sativum* cv. Kleine Rheinländerin	2.96	1.4
Pop. 47	1	1	**4**	0.58	1.16	2.33	*Raphanus sativus*	4.14	3.17
Pop. 47	3	1	**4**	0.57	1.13	2.27	*Raphanus sativus*	3.26	2.47
Pop. 47	2;4->7	5	**4**	0.56	1.12	2.23	*Raphanus sativus*	4.12	3.14
Pop. 47	8->12	5	**4**	0.56	1.13	2.25	*Raphanus sativus*	3.67	3.28
Pop. 47	13->17	5	**4**	0.56	1.11	2.23	*Raphanus sativus*	3.62	3.62
Pop. 47	18->20	3	**4**	0.56	1.13	2.25	*Raphanus sativus*	4.39	4.93
Pop. 48	1,4,6,7,10	5	**6**	0.69	2.06	4.12	*Solanum lycopersicum*	4.58	2.71
Pop. 48	2	1	**6**	0.64	1.92	3.84	*Solanum pseudocapsicum*	6.64	4.35
Pop. 48	3	1	**6**	0.61	1.84	3.68	*Pisum sativum* cv. Kleine Rheinländerin	4.32	3.25
Pop. 48	5	1	**6**	0.63	1.90	3.81	*Zea mays* cv. CE-777	3.46	1.5
Pop. 48	8	1	**6**	0.63	1.88	3.76	*Zea mays* cv. CE-777	4.90	1.39
Pop. 48	9	1	**6**	0.62	1.85	3.70	*Pisum sativum* cv. Kleine Rheinländerin	4.97	3.67
Pop. 49	1	1	**6**	0.63	1.89	3.78	*Zea mays* cv. CE-777	3.64	1.54
Pop. 49	2,4,5	3	**6**	0.61	1.83	3.66	*Solanum lycopersicum*	5.14	3.68
Pop. 49	3	1	**6**	0.59	1.78	3.57	*Pisum sativum* cv. Kleine Rheinländerin	4.47	3.25
Pop. 49	6	1	**6**	0.62	1.85	3.70	*Zea mays* cv. CE-777	5.49	1.22
Pop. 49	7->10	4	**6**	0.68	2.03	4.05	*Solanum lycopersicum*	4.27	3.15
Pop. 50	1	1	**6**	0.65	1.96	3.92	*Solanum lycopersicum*	5.81	3.02
Pop. 50	2;5->7	4	**6**	0.65	1.96	3.93	*Solanum lycopersicum*	4.60	2.97
Pop. 50	3	1	**6**	0.64	1.91	3.82	*Zea mays* cv. CE-777	3.80	1.92

**Population**	**Individual/s**	**no. of inds.**	**Ploidy**	**1Cx**	**1C**	**2C**	**Standard**	**CV sample**	**CV standard**
Pop. 50	4	1	**6**	0.62	1.86	3.72	*Pisum sativum* cv. Kleine Rheinländerin	5.30	1.74
Pop. 50	8	1	**6**	0.63	1.89	3.78	*Zea mays* cv. CE-777	6.37	1.75
Pop. 50	9->11	3	**6**	0.66	1.98	3.96	*Solanum lycopersicum*	4.86	3.43

Table S4. Contribution of environmental variables to the first two principal components (PC1 and PC2) of the PCA-env.

Variable	PC1	PC2
**Alt**	18.41	14.65
**Bio.1**	3.07	40.40
**Bio.3**	4.51	4.43
**Bio.4**	23.29	0.95
**Bio.8**	24.88	1.26
**Bio.9**	3.85	31.52
**Bio.15**	3.66	3.65
**Bio.19**	18.29	3.09

Table S5. The percentage of niche overlap and the components of climatic niche dynamics (expansion, stability and unfilling) are shown for each comparison.

1	2	Niche Overlap (%)	Expansion (E)	Stability (S)	Unfilling (U)
2x	6x	11.18%	0.782	0.218	0.408
4x	6x	20.38%	0.605	0.395	0.167
2x	4x	38.76%	0.295	0.705	0.238
